# Apolipoprotein E *ε*4 Polymorphism as a Risk Factor for Ischemic Stroke: A Systematic Review and Meta-Analysis

**DOI:** 10.1155/2022/1407183

**Published:** 2022-02-03

**Authors:** Su-Ya Qiao, Ke Shang, Yun-Hui Chu, Hai-Han Yu, Xin Chen, Chuan Qin, Deng-Ji Pan, Dai-Shi Tian

**Affiliations:** Department of Neurology, Tongji Hospital, Tongji Medical College, Huazhong University of Science and Technology, Wuhan 430030, China

## Abstract

**Introduction:**

Rising studies indicate that the apolipoprotein E (APOE) gene is related to the susceptibility of ischemic stroke (IS). However, certain consensus is limited by the lack of a large sample size of researches. This meta-analysis was performed to explore the potential association between the APOE gene and IS.

**Methods:**

To identify relevant case control studies in English publications by October 2020, we searched PubMed, Embase, Web of Science, and the Cochrane Library. Pooled odds ratios (ORs) with fixed- or random-effect models and corresponding 95% confidence intervals (CIs) were calculated to analyze potential associations.

**Results:**

A total of 55 researches from 32 countries containing 12207 IS cases and 27742 controls were included. The association between APOE gene *ε*4 mutation and IS was confirmed (*ε*4 vs. *ε*3 allele: pooled OR = 1.374, 95% CI, 1.214-1.556; *ε*2/*ε*4 vs. *ε*3/*ε*3: pooled OR = 1.233, 95% CI, 1.056-1.440; *ε*3/*ε*4 vs. *ε*3/*ε*3: pooled OR = 1.340, 95% CI, 1.165-1.542; *ε*4/*ε*4 vs. *ε*3/*ε*3: pooled OR = 1.833, 95% CI, 1.542-2.179; and APOE *ε*4 carriers vs. non-*ε*4 carriers: pooled OR = 1.377; 95% CI, 1.203-1.576). Interestingly, APOE *ε*4 mutation showed a dose-response correlation with IS risk (*ε*4/*ε*4 vs. *ε*2/*ε*4: pooled OR = 1.625; 95% CI, 1.281-2.060; *ε*4/*ε*4 vs. *ε*3/*ε*4: pooled OR = 1.301; 95% CI, 1.077-1.571). Similar conclusions were drawn in the small artery disease (SAD) subtype, but not in large artery atherosclerosis (LAA) or in cardioaortic embolism (CE), by subgroup analysis.

**Conclusions:**

These observations reveal that specific APOE *ε*4 mutation was significantly associated with the risk of IS in a dose-dependent manner, while APOE *ε*4 mutation was related to SAD subtype onset without a cumulative effect.

## 1. Introduction

Ischemic stroke (IS) is a disturbing problem worldwide, which is attributable to its leading role in disability and mortality worldwide, regardless of age, ethnicity, or gender [1]. Uncovering the etiology of IS is crucial for recognition and prevention of this disorder. Genetic elements and environmental components positively contribute to this multifactorial disease [2, 3]. Genetic inheritance provides a guide to the identification of high-risk individual. It deserves to investigate candidate gene polymorphisms in IS pathophysiological pathways. The apolipoprotein E (APOE) gene locates on chromosome 19q13.2. Two single polymorphisms (rs7412 and rs729358), three common alleles (*ε*2, *ε*3, and *ε*4), and six genotypes (*ε*2/*ε*2, *ε*2/*ε*3, *ε*2/*ε*4, *ε*3/*ε*3, *ε*3/*ε*4, and *ε*4/*ε*4) generate in populations [4]. The product of the APOE gene is a polymorphic protein named apolipoprotein E, which modulates the translocation of the cholesterol and other lipids among highly diverse cells [5], involved with neuroinflammation [6] and myelin integrity maintenance [7]. A study indicated that the activated CypA–MMP9 pathway in APOE4 carriers facilitated pericyte injury, which caused blood vessel dysfunction [8]. APOE polymorphisms and its risk associations with coronary artery disease [9], hypertension [10], diabetes [11], and carotid arterial atherosclerosis [12] are widely debated. The abovementioned diseases place individuals at a potential serious risk of IS. Individual studies of the association between IS and APOE polymorphisms have been explored extensively. Clinical differences, ethnic diversities, and small sample sizes restricted the present finding to an inconsistent and controversial one. Previous meta-analyses concerning to this issue have been published several years ago [13] or limited to specific ethnicity [14, 15]. Accordingly, researches from 32 countries are qualified to form our meta-analysis to clarify how APOE genotypes are associated with IS. Moreover, we firstly revealed the correlation of the APOE gene and three IS subtypes (large artery atherosclerosis (LAA), small artery disease (SAD), and cardioaortic embolism (CE)).

## 2. Materials and Methods

We followed the rules of the preferred reporting items for systematic reviews and meta-analyses (PRISMA) statement to make this meta-analysis [16].

### 2.1. Data Availability

The data that contribute to the findings in our study are available and the corresponding authors can be contacted for data access.

### 2.2. Literature Search

Online databases (PubMed, Embase, Web of Science, and the Cochrane Library) were comprehensively searched for studies potentially involved and published in English publications and prior to October 30, 2020. We used a combination of some search terms relevant for IS (stroke, cerebral infarct, brain infarct, ischemic stroke, cerebral ischemia, transient ischemic attack, and cerebrovascular accident) and for the APOE gene (apolipoprotein E, APOE polymorphisms, apolipoprotein E polymorphisms, apolipoprotein E gene, rs429358, rs7412, apolipoprotein E epsilon 4, APOE e4, apolipoprotein E epsilon 2, and APOE e2). The detailed search strategies were showed next.

### 2.3. Selection Criteria

The selection of the studies was independently completed by two investigators, and any difference was resolved by discussion until an agreement was reached. We carefully selected case control studies that evaluated the relationship of the APOE gene and IS with definite IS diagnoses (using computed tomography, magnetic resonance, or autopsy) regardless of the ethnic background. The detailed inclusion criteria were (1) high-quality studies which explore the relationship between the APOE gene and IS, (2) explicit IS diagnostic criteria, (3) nonstroke individuals as the control group, and (4) original data including independent and sufficient APOE genotype data, to compute ORs and 95% CIs. The newest and largest studies were chosen to avoid duplicate or overlapped data information.

### 2.4. Data Extraction

Two investigators separately finished full-text reading to extract the needed information from each selected study and resolved the controversial items through serious discussion. The extracted information was (1) research characteristics, including the first author's name, year of publication, and geographical location of the study; (2) participant details, such as the sex ratio, mean age, and the sample size of case and control groups; (3) diagnostic criteria for IS; (4) determination methods of the APOE gene; (5) each genotype frequency; (6) the sample sizes of IS subtypes according to TOAST norms and respective genotype frequency; and (7) HWE in controls.

### 2.5. Quality Assessment

We performed the quality assessment through the Newcastle-Ottawa Scale (NOS) score considering selection, comparability, and exposure. It ranged from 0 (worst) to 9 (best) and high-quality studies were known as with a NOS score ≥ 7.

### 2.6. Statistical Analysis

We performed Stata 14.0 to complete all data analyses. The chi-square test was used to examine the Hardy-Weinberg equilibrium (HWE) in control groups. An overt deviation from HWE was regarded as *P* < 0.05. The compositive ORs and 95% CI were calculated. We explored five genetic models to generate the respective pooled ORs: (1) allele comparisons (*ε*2 allele vs. *ε*3 allele; *ε*4 allele vs. *ε*3 allele); (2) genotype comparisons (*ε*2/*ε*2 vs. *ε*3/*ε*3; *ε*2/*ε*3 vs. *ε*3/*ε*3; *ε*2/*ε*4 vs. *ε*3/*ε*3; *ε*3/*ε*4 vs. *ε*3/*ε*3; *ε*4/*ε*4 vs. *ε*3/*ε*3); (3) APOE *ε*4 carrier comparisons: we defined three *ε*4-containing genotypes (*ε*2/*ε*4 + *ε*3/*ε*4 + *ε*4/*ε*4) as APOE *ε*4 carriers and the other genotypes (*ε*2/*ε*2 + *ε*2/*ε*3 + *ε*3/*ε*3) as non-APOE *ε*4 carriers; (4) APOE *ε*2 carrier comparisons: similar comparisons of *ε*2-containing genotypes (*ε*2/*ε*2 + *ε*2/*ε*3 + *ε*2/*ε*4) vs. non-*ε*2-containing genotypes (*ε*3/*ε*3 + *ε*3/*ε*4 + *ε*4/*ε*4); and (5) comparisons between APOE *ε*4 homozygosis and *ε*4 heterozygote (*ε*4/*ε*4 vs. *ε*2/*ε*4; *ε*4/*ε*4 vs. *ε*3/*ε*4). The *I*^2^ statistic and Cochran's *Q* test were applied to measure the heterogeneity between studies [17]. We selected the random effect model (DerSimonian-Laird method) when heterogeneity was found between studies (*I*^2^ > 50.0%) and fixed-effect model (Mantel-Haenszel method) when no heterogeneity existed (*I*^2^ < 50.0%). Subgroup analysis was conducted to confirm the relationship between the APOE polymorphisms and the risk of different IS subgroups. Sensitivity analysis was performed by successively removing a single study one by one to verify the stability and reliability of our conclusions. Meta-regression analysis was operated to recognize sources of heterogeneity. Funnel plots and quantified Egger's tests were accomplished to test publication bias. Significant publication bias was considered as the *P* value of Egger's test less than 0.10 or obvious asymmetric funnel plot.

### 2.7. The Result of Trial Sequential Analysis (TSA)

Insufficient sample size, continuous updating, and repeating “ significance testing” could increase the risk of type I errors. Therefore, traditional meta-analysis that focuses on the specific topic may suffer an increased risk of random error. Trial sequential analysis (TSA) was used to reduce the risk of type I error and obtain important information regarding the required sample size for such trials. Set the time sequence of a single study as the research node, and then, perform an interim analysis between the new study that will be included in meta-analysis and existing data accumulation. The required information size (RIS), trial sequential monitoring boundary, and futility boundary are estimated using the TSA. As the sample size of meta-analysis reaching the RIS or the *z*-curve crossing the trial sequential monitoring boundary, we can conclude that the results of meta-analysis are quite stable and further studies were not needed. We accomplished TSA following the guidelines of the user manual and previous article [18] by setting a significance of 5% for type I error, a relative risk reduction of 20%, and a statistical test power of 80% with TSA software (TSA, version 0.9 beta; Copenhagen Trial Unit, Copenhagen, Denmark).

## 3. Results

### 3.1. Characteristics of Eligible Studies

**Figure 1:**
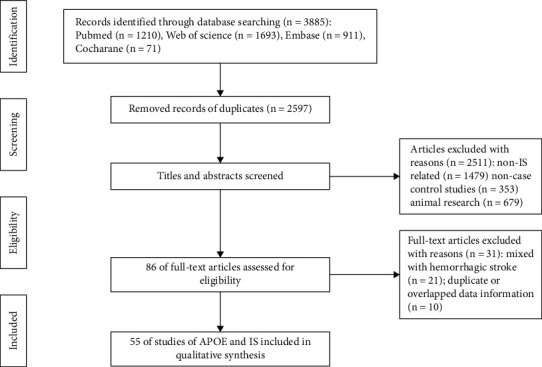
A flow diagram of identification and selection process of the included literatures in this meta-analysis.

We collect a total of 55 studies from 32 countries containing 12207 IS cases and 27742 controls to make the meta-analysis [19–73].  Figure 1 showed the detailed selection process. The selected studies and their main characteristics were exhibited in Table 1. Fifteen of the studies provided data about different subtypes (grouped by classification of cerebrovascular diseases III or TOAST classification) of IS: large artery atherosclerosis (LAA), small artery disease (SAD), and cardioaortic embolism (CE). We extracted them independently and specific information was showed in supplementary material table 1. There were seven studies (Koopal et al. 2016, Lai et al. 2007, Chowdhury et al. 2001, Kokubo et al. 2000, Ji et al. 1998, Couderc et al. 1993, Saidi et al. 2009) which deviated HWE obviously, and one study (Schneider et al. 2005) did not contain enough data to obtain HWE. Forty-eight studies used PCR-based method and seven researches (Slowik et al. 2003, Karttunen et al. 2002, Hachinski et al. 1996, Couderc et al. 1993, Brewin et al. 2020, Aalto-Setala et al. 1998, Schneider et al. 2005) used other methods to identify APOE genotypes. These studies used computed tomography or magnetic resonance to diagnose IS except that one research which used autopsy (Schneider et al. 2005). The NOS score mean value was 7.509, which suggested that the quality of included studies was reliable (supplementary material Table 2). PRISMA2020 checklist was provided to present our meta-analysis items (supplementary material Table 3).

### 3.2. Main Results of the Comparisons in the Abovementioned Five Genetic Models

#### 3.2.1. Allele Comparisons

**Figure 2:**
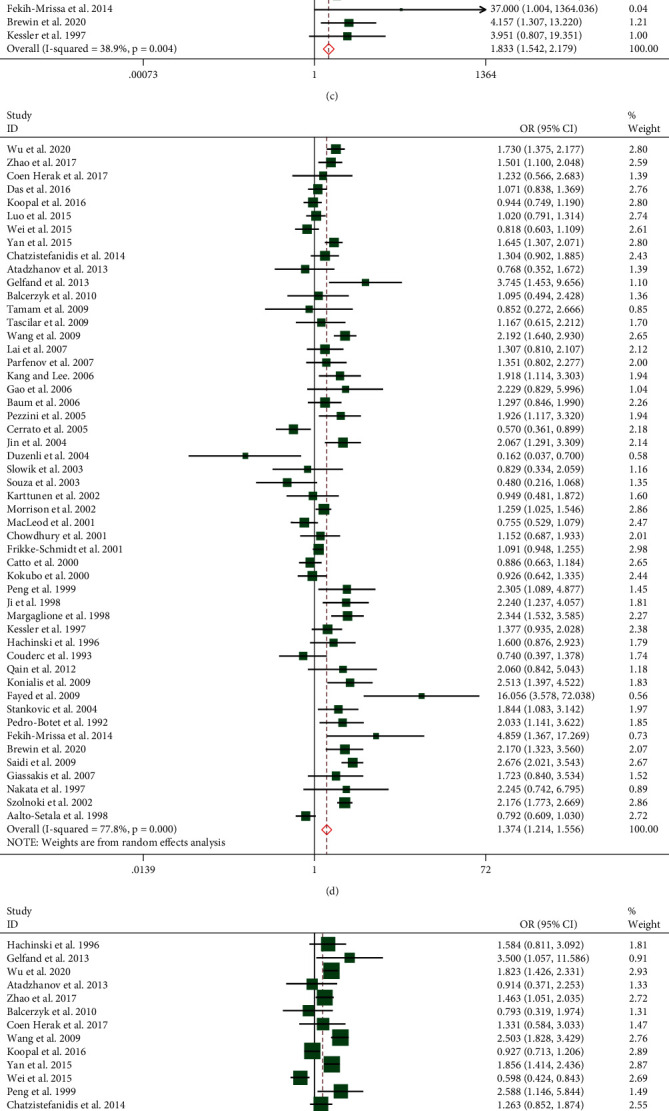
(a–g) Forest plots of the relationships between APOE gene polymorphisms in all studies included. (a) Forest plot of *ε*2/*ε*4 vs. *ε*3/*ε*3 comparison. (b) Forest plot of *ε*3/*ε*4 vs. *ε*3/*ε*3 comparison. (c) Forest plot of APOE *ε*4/*ε*4 vs. the *ε*3/*ε*3 genotype. (d) Forest plot of the APOE *ε*4 allele vs. *ε*3 allele. (e) Forest plot of APOE *ε*4 carriers vs. non-*ε*4 carriers. (f) Forest plot of APOE *ε*4/*ε*4 vs. *ε*2/*ε*4. (g) Forest plot of APOE *ε*4/*ε*4 vs. *ε*3/*ε*4.

In comparison with the *ε*3 allele, the *ε*2 allele did not show association of the risk of IS (pooled OR = 0.983, 95% CI, 0.867-1.115, *P* = 0.79) (as showed in Table 2), while the *ε*4 allele contributed to an obviously increased risk of IS with the pooled OR = 1.374 (95% CI, 1.214-1.556, *P* < 0.0001) (Figure 2(d)).

#### 3.2.2. Genotype Comparisons

When compared with the *ε*3/*ε*3 genotype, the pooled effects of the APOE genotype in the meta-analysis were as follows: for the *ε*2/*ε*2 genotype, pooled OR = 0.985, 95% CI, 0.653-1.486, *P* = 0.94, and for the *ε*2/*ε*3 genotype, pooled OR = 0.980, 95% CI, 0.900-1.066, *P* = 0.63; those two genotypes presented no association with the risk of IS (as showed in Table 2). Genotypes *ε*2/*ε*4, *ε*3/*ε*4, and *ε*4/*ε*4 were related to a higher risk of IS than *ε*3/*ε*3. The respective IS risk ORs were 1.233 (95% CI, 1.056-1.440, *P* = 0.01) (Figure 2(a)), 1.340 (95% CI, 1.165-1.542, *P* < 0.0001) (Figure 2(b)), and 1.833 (95% CI, 1.542-2.179, *P* < 0.0001) (Figure 2(c)). The above results could be found in Table 2. A conclusion was drawn: every genotype which contained APOE *ε*4 mutation increased the risk of IS.

#### 3.2.3. APOE *ε*4 Carrier Comparisons

Compared with the non-*ε*4 carriers, we confirmed that the *ε*4 carriers were associated with the increased risk of IS; the pooled outcome was pooled OR = 1.377 (95% CI, 1.203-1.576, *P* < 0.0001) (Figure 2(e)).

#### 3.2.4. APOE *ε*2 Carrier Comparisons

In the genetic model of *ε*2 carriers vs. non-*ε*2 carriers, there was no association with the IS risk (pooled OR = 0.956, 95% CI 0.841-1.086, *P* = 0.49) (Table 2).

#### 3.2.5. APOE *ε*4 Homozygosis versus APOE *ε*4 Heterozygote Comparisons

Given the above, the APOE *ε*4 mutation was linked to IS risk. To identify whether there is a dose-response relationship between the *ε*4 allele and IS or not, we implemented the comparisons between the *ε*4/*ε*4 genotype and *ε*4 heterozygotes (*ε*2/*ε*4 or *ε*3/*ε*4 genotype). Compared with the *ε*2/*ε*4 and *ε*3/*ε*4 genotypes, the IS risk ORs for *ε*4/*ε*4 genotypes were 1.625 (95% CI, 1.281-2.060, *P* < 0.0001) and 1.301 (95% CI, 1.077-1.571, *P* = 0.01), respectively (Figures 2(f) and 2(g)); this part provided evidence that *ε*4 homozygosis might generate a higher risk of IS than *ε*4 heterozygotes.

### 3.3. Main Results of the Relationship between APOE Gene and Three IS Subtypes

**Figure 3:**
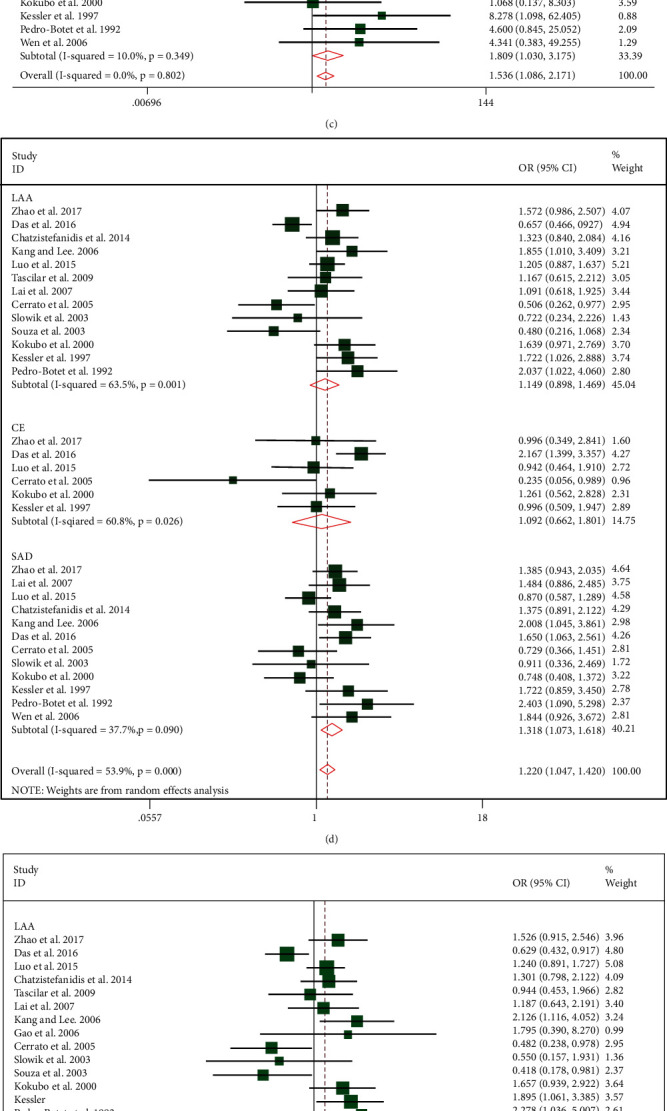
(a–g) Forest plots of the relationships between APOE gene polymorphisms in subgroup analysis. (a) Forest plot of *ε*2/*ε*4 vs. *ε*3/*ε*3 comparison. (b) Forest plot of *ε*3/*ε*4 vs. *ε*3/*ε*3 comparison. (c) Forest plot of APOE *ε*4/*ε*4 vs. the *ε*3/*ε*3 genotype. (d) Forest plot of the APOE *ε*4 allele vs. *ε*3 allele. (e) Forest plot of APOE *ε*4 carriers vs. non-*ε*4 carriers. (f) Forest plot of APOE *ε*4/*ε*4 vs. *ε*2/*ε*4. (g) Forest plot of APOE *ε*4/*ε*4 vs. *ε*3/*ε*4.

We further investigated on the correlation of APOE gene polymorphisms and risks of IS subtypes by making comparisons in five genetic models, with a particular focus on the APOE *ε*4 mutation. Subgroup analyses showed that APOE *ε*4 mutation significantly increased SAD risk (*ε*4 allele vs. *ε*3 allele: pooled OR = 1.318, 95% CI, 1.073-1.618, *P* = 0.01 (Figure 3(d)); *ε*3/*ε*4 vs.*ε*3/*ε*3: pooled OR = 1.392, 95% CI, 1.097-1.767, *P* = 0.01 (Figure 3(b)); *ε*4/*ε*4 vs. *ε*3/*ε*3: pooled OR = 1.809, 95%, CI 1.030-3.175, *P* = 0.04 (Figure 3(c)); and APOE *ε*4 carriers vs. non-APOE *ε*4 carriers: pooled OR = 1.329, 95% CI, 1.064-1.661, *P* = 0.01 (Figure 3(e))). But genotype *ε*2/*ε*4 did not increase the risk of SAD onset (Figure 3(a)). The result of APOE *ε*4 homozygosis versus *ε*4 heterozygote comparisons (*ε*4/*ε*4 vs. *ε*2/*ε*4 and *ε*4/*ε*4 vs. *ε*3/*ε*4) was a matter of concern: APOE *ε*4 mutation could not cause a cumulative effect in generating higher risk of SAD onset, as showed in Figures 3(f) and 3(g).

### 3.4. Sensitivity Analysis

Sensitivity analysis was performed by removing studies one by one to check the effect of the individual study on overall ORs. No single study influenced on the pooled ORs and 95% CIs in all genetic model comparisons as our data showed (supplementary material table 4).

### 3.5. Publication Bias

We carried out publication bias analysis by using funnel plots as qualitative description and Egger's regression tests as quantitative outcome. Funnel plots of all genetic model comparisons did not exhibit apparent asymmetry (several funnel plots were showed in supplementary material figure 1 and 2). In addition to subtype analysis of *ε*2/*ε*2 vs. *ε*3/3, all the Egger's regression test outcomes indicated that there existed no evident publication bias with all *P* values exceeding 0.1 (supplementary material table 5). The above results showed that publication bias of our meta-analysis was not significant.

### 3.6. Regression Analysis

Meta-regression analysis was then performed to explore sources of heterogeneity as shown in supplementary material table 5, considering the year of publication, region, sample size, genotyping method, HWE, NOS score, and source of control. However, the *P* value of each factor affecting overall heterogeneity was not statistically significant in comparisons of *ε*3/*ε*4 vs. *ε*3/3, *ε*4 vs. non-*ε*4, *ε*2 vs. non-*ε*2, *ε*4allele vs. *ε*3allele, and *ε*2allele vs. *ε*3allele (supplementary material figure 3). Heterogeneity sources were unascertainable.

### 3.7. The Result of Trial Sequential Analysis (TSA)

**Figure 4:**
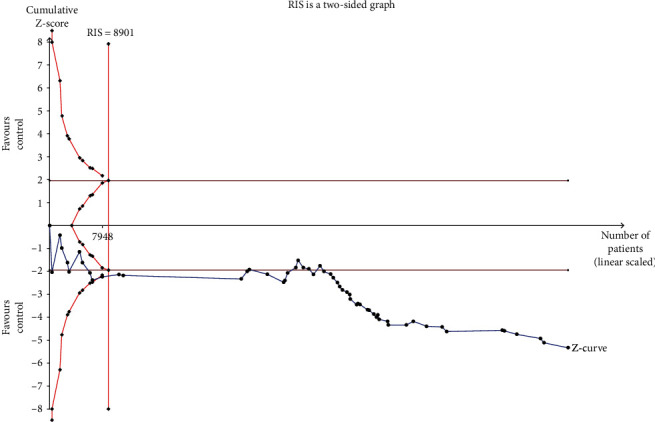
Trial sequential analysis of the association between APOE gene polymorphisms and ischemic stroke.

The RIS was 8901 samples and the sample size of our meta-analysis reached it. Moreover, the cumulative *z*-curve crossed the trial sequential monitoring boundary before reaching the RIS as showed in Figure 4. The result of TSA guaranteed the stability of our meta-analysis results. Our sample size was proved to be enough for evaluating the relationship between APOE polymorphisms and IS risk.

## 4. Discussion

Recently, scholars explored more how gene polymorphisms were contributing to the occurrence and prognosis of diseases. And several previous publications had well explored how gene polymorphisms related to diseases onset and potential mechanisms [74, 75]. As a heterogeneous multifactorial disorder, ischemic stroke could be regulated by certain gene synthesis and specific gene products. The genes involved in the pathological process of stroke are also worth of attention. Apolipoprotein E has been proven to affect atherosclerosis, neurodegeneration, and the process of nerve damage repair. That is why we explored the relationship between APOE gene polymorphisms and ischemic stroke risk.

APOE is a 299-amino acid protein encoded by the APOE gene of three common polymorphisms, *ε*2, *ε*3, and *ε*4. The correlation of APOE gene polymorphisms and the risk of cerebral vascular and degenerative diseases have been investigated a lot, especially in Alzheimer's disease (AD) and cerebral amyloid angiopathy (CAA) [76]. APOE *ε*4 is associated with increased risk for AD whereas APOE *ε*2 is associated with decreased risk [77]. Mirza et al. performed a meta-analysis to find that greater WMH volume was associated with worse performance on all cognitive domains in APOE *ε*4 carriers only in AD [78]. Charidimou et al. proved that the APOE *ε*2 allele might be associated with the pathophysiology and severity of cortical superficial siderosis in CAA [79]. As to IS, there existed quite many researches with inconsistent conclusions. Besides method differences, ethnic difference and unclarified pathophysiological mechanisms are probable reasons of the inconsistency.

In a meta-analysis in 1999, McCarron et al. found that the *ε*4 allele and carriers were more frequent among patients with ischemic cerebrovascular disease, compared with control subjects (27% versus 18%; odds ratio, 1.73; 95% CI, 1.34-2.23; *P* < 0.0001) [13]. In another meta-analysis based on Chinese population, the *ε*4 allele is associated with an increased risk of developing cerebral infarction, in which the adjusted risk estimate for the *ε*4 allele versus *ε*3 allele was significant (OR = 2.00, 95% CI 1.59-2.53, *P* < 0.0001) [14]. Our estimates seemed to be coinciding with the above ones. Compared with the *ε*3 allele, the *ε*4 allele showed a higher risk of IS. Compared with *ε*3/*ε*3, both *ε*4 heterozygote (*ε*2/*ε*4, *ε*3/*ε*4) and *ε*4 homozygosis (*ε*4/*ε*4) exhibited a significant correlation with an increased risk of IS. Notably, OR in *ε*4 homozygosis (*ε*4/*ε*4 vs. *ε*3/3: 1.833 (95% CI 1.542-2.179)) was higher than those in *ε*4 heterozygotes (*ε*2/*ε*4 vs. *ε*3/3: 1.233 (95% CI 1.056-1.440) and *ε*3/*ε*4 vs. *ε*3/3: 1.340 (95% CI 1.165-1.542)), which implied that the *ε*4 allele might possess a cumulative effect. Then, we performed comparisons between *ε*4/*ε*4 and *ε*2/*ε*4 or *ε*3/*ε*4; there existed significant differences between *ε*4 homozygosis and *ε*4 heterozygote. The OR between *ε*4/*ε*4 and *ε*2/*ε*4 was 1.625 (95% CI 1.281-2.060, *P* < 0.0001); the OR between *ε*4/*ε*4 and *ε*3/*ε*4 was 1.301 (95% CI 1.077-1.571, *P* = 0.01), giving a hint that *ε*4 homozygosis might bring a higher risk of IS than *ε*4 heterozygotes.

There are tremendous researches and discussions focusing on the pathogenicity of *ε*4. An Indian research reported that VLDL and triglycerides levels were found to be significantly associated with *ε*2/*ε*4 and *ε*3/*ε*4 genotypes; the *ε*4 allele exerted a higher influence than the *ε*3 allele in plasma cholesterol levels [22]. As a lipid transport protein, APOE3 and APOE2 preferentially bind to the smaller, more phospholipid-enriched high-density lipoproteins (HDL), while APOE4 preferentially binds to the larger, triglyceride-rich very low-density lipoproteins (VLDL). Miyata and Smith demonstrated an antioxidant activity in the order APOE2 > E3 > E4, and other researchers also reported similar results that APOE4 was associated with increased oxidative stress [25, 80], which might play a role in atherosclerosis and lead to increased risk of ischemic vascular diseases. Besides the above reasons, APOE4 was proved to be neurotoxic by assuming an abnormal conformation (the unique domain interaction between Arg-61 and Glu-255) which was highly susceptible to neuron specific proteolysis and generating neurotoxic fragments that escaped the secretory pathway and entered the cytosol [81]. Totally, from pathophysiological mechanisms to clinical research results, it seems that APOE4 is indeed related to a higher risk of IS, compared with other isoforms, both in *ε*4 heterozygote and homozygous. *ε*2 allele appears to be unclear and controversial in stroke [13]. In a meta-analysis of Martínez-González et al., compared with *ε*3/*ε*3, APOE *ε*2 was associated with intracerebral hemorrhage (OR = 1.32; 95% CI, 1.01-1.74); meanwhile, APOE *ε*2 was more related to lobar hemorrhage than deep hemorrhage [82]. As to the association of IS with APOE based on previous investigation, it is uncertain. Our estimates showed that both *ε*2/*ε*2 and *ε*2/*ε*3 genotypes exhibited no significant effects on IS risk, compared with *ε*3/*ε*3. Also, no differences were found in comparisons of *ε*2 allele vs. *ε*3 allele and *ε*2 vs. non-*ε*2 carriers. This result remained consistent with another meta-analysis in 2013 [14]. Interestingly, in subtype analysis, *ε*2/*ε*2 displayed significances in the CE group (OR = 4.290; 95% CI, 1.917-9.600; *P* < 0.0001) and SAD group (OR = 1.803; 95% CI, 1.037-3.134; *P* = 0.04). The largest meta-analysis of the APOE genotype with IS showed a positive linear association of increasing risk when ordered from *ε*2/*ε*2, *ε*2/*ε*3, *ε*2/*ε*4, *ε*3/*ε*3, *ε*3/*ε*4, and *ε*4/*ε*4 in European ancestry population [83]. The conclusion might explain why APOE4 brings a higher risk of IS but could not clarify that the CE and SAD subgroups in comparison of *ε*2/*ε*2 with *ε*3/*ε*3 show significances. It is well known that all patients with type III hyperlipidemia (dysbetalipoproteinemia) were APOE *ε*2 homozygous, whereas most *ε*2/*ε*2 subjects (>90%) were normolipidemic or even hypolipidemic, owing to reductions in LDL or HDL or both. Therefore, the APOE *ε*2 allele has both increased and decreased risks for atherosclerosis, which induced a comprehensive and undetermined result [84].

As to our subtype analyses, all LAA groups showed no significant difference among comparisons, which raised a question why isoforms of APOE, a lipid transport protein, seemed not to be related with IS caused by large artery atherosclerosis. Besides lipid metabolism and atherosclerosis, there might exist some other pathways underlying the relationships between APOE and risk of IS. Our estimates displayed that APOE isoforms were associated to risk of IS especially in the SAD subgroup. Hypertension was known to be an independent risk factor of SAD. Atherosclerosis, dyslipidemia, and hypertension have a complex interaction, and the causations with APOE need further investigation.

Our meta-analysis has several limitations. First, just as the abovementioned, heterogeneity between studies remains undeterminable. Second, results of our meta-analysis based on case control studies cannot provide a causal relationship, but only an association. Third, age variable and ethnicity can influence APOE frequencies in a population; we cannot obtain sufficient related information to perform further subdivided subgroup analyses. Fourth, other pathogenic factors about IS, a multifactorial disease, such as plasma lipid levels, hypertension, life-style, BMI, and gene-environment interactions, were unachievable. Fifth, the controls in accessible studies were not strictly defined; some were selected from healthy populations and others were from nonstroke people. The expected genotype distribution in controls was not in accordance with HWE in seven studies. Population selection in control groups failed to avoid certain diseases which might have a relation with the APOE gene, such as dyslipidemia, hypertension, other vascular diseases, and diabetes. Sixth, the case groups were not selected by a prospective process and the design of case control studies often caused abnormal gene frequency.

## 5. Conclusions

In conclusion, our meta-analysis provides rational evidence that APOE *ε*4 mutation is a genetic risk factor for IS. Prospective studies of a large sample size, which concerns gene-gene and gene-environment interactions, should be carried out in the future to reach a more comprehensive outcome about the association of APOE gene polymorphisms and IS. What is more, future researches should be designed to elucidate the mechanism by which APOE *ε*4 mutation adds the risk of IS.

## Figures and Tables

**Table 1:** Main characteristics of studies associated with APOE polymorphisms and IS stroke included in this meta-analysis.

Study ID	Region	Criteria for IS	Genotyping method	Source of control	Characteristics and the counts of every genotype													*H*	*N*
					Group	Sample size	Male/*n* (%)	Age(years)	*ε*2/*ε*2	*ε*2/*ε*3	*ε*2/*ε*4	*ε*3/*ε*3	*ε*3/*ε*4	*ε*4/*ε*4	*ε*2 allele	*ε*3 allele	*ε*4 allele		
Wu et al., 2020 [19]	China	CT/MRI	PCR	H-B	Case	938	581 (61.9%)	65.6 ± 10.6	2	63	18	684	156	15	85	1587	204	Y	8
					Control	1028	622 (60.5%)	63.7 ± 12.4	9	131	13	763	106	6	162	1763	131		

Zhao et al., 2017 [20]	China	CT/MRI	PCR	H-B	Case	513	294 (57.3%)	62.3 ± 12.2	3	63	7	347	85	8	76	842	108	Y	7
					Control	514	288 (56.0%)	61.7 ± 13.5	5	70	8	366	64	1	88	866	74		

Coen Herak et al., 2017 [21]	Croatia	CT/MRI	PCR	P-B	Case	73	48 (65.8%)	4.3 ± *X*	0	10	2	50	11	0	12	121	13	Y	8
					Control	100	63 (63.0%)	6.5 ± *X*	1	11	0	74	13	1	13	172	15		

Das et al., 2016 [22]	Indian	CT/MRI	PCR-RFLP	P-B	Case	620	434 (70.0%)	49.4 ± 17.4	5	46	6	431	120	12	62	1028	150	Y	8
					Control	620	428 (69.0%)	49.1 ± 16.9	5	50	4	436	113	12	64	1035	141		

Koopal et al., 2016 [23]	Netherlands	CT	PCR	P-B	Case	278	NA	NA	3	30	8	160	69	8	44	419	93	N	7
					Control	4220	NA	NA	50	389	96	2422	1127	136	585	6360	1495		

Luo et al., 2015 [24]	China	CT/MRI	PCR	H-B	Case	712	465 (65.3%)	65.2 ± 13.9	4	93	13	494	101	7	114	1182	128	Y	7
					Control	774	418 (54.0%)	51.5 ± 16.9	3	107	8	535	113	8	121	1290	137		

Wei et al., 2015 [25]	Malaysia	CT/MRI	PCR	P-B	Case	297	33 (11.1%)	52.6 ± 8.8	8	68	23	137	54	7	107	396	91	Y	8
					Control	297	119 (40.0%)	51.8 ± 8.7	4	12	27	163	89	2	47	427	120		

Yan et al., 2015 [26]	China	CT/MRI	PCR-RFLP	H-B	Case	580	387 (66.7%)	59.8 ± 13.7	11	41	33	351	82	62	96	825	239	Y	8
					Control	580	379 (65.3%)	59.4 ± 13.1	61	54	49	354	33	29	225	795	140		

Chatzistefanidis et al., 2014 [27]	Greece	CT/MRI	PCR	H-B	Case	329	225 (68.4%)	59.7 ± 11.6	3	36	3	227	56	4	45	546	67	Y	7
					Control	361	205 (56.8%)	60.4 ± 13.7	2	24	8	278	47	2	36	627	59		
Atadzhanov et al., 2013 [28]	Zambian	CT	PCR	P-B	Case	23	NA	54.0 ± 16.0	0	4	3	9	7	0	7	29	10	Y	9
					Control	116	50 (41.4%)	NA	0	25	7	38	37	9	32	138	62		

Gelfand et al., 2013 [29]	America	CT/MRI	PCR-RFLP	H-B	Case	13	10 (77.0%)	NA	0	1	2	5	3	2	3	14	9	Y	8
					Control	84	46 (55.0%)	NA	0	8	3	55	16	2	11	134	23		

Balcerzyk et al., 2010 [30]	Poland	CT/MRI	PCR	P-B	Case	72	42 (58.3%)	8.8 ± 5.6	1	9	0	52	6	4	11	119	14	Y	7
					Control	71	41 (57.8%)	8.2 ± 5.4	0	8	0	51	11	1	8	121	13		

Tamam et al., 2009 [31]	Turkey	CT/MRI	PCR	H-B	Case	65	NA	NA	0	7	2	50	5	1	9	112	9	Y	7
					Control	30	10 (33.3%)	61.9 ± 14.7	0	1	1	25	2	1	2	53	5		

Tascilar et al., 2009 [32]	Turkey	CT/MRI	PCR	P-B	Case	85	51 (60.0%)	61.7 ± 13.6	3	18	3	45	9	7	27	117	26	Y	7
					Control	77	25 (32.5%)	54.7 ± 8.4	3	16	7	40	9	2	29	105	20		

Wang et al., 2009 [33]	China	CT/MRI	PCR	H-B	Case	396	209 (52.8%)	57.3 ± 8.2	16	98	60	124	87	11	190	433	169	N	7
					Control	396	202 (51.0%)	57.3 ± 8.1	33	116	41	164	39	3	223	483	86		

Lai et al., 2007 [34]	China	MRI	PCR	H-B	Case	257	164 (63.8%)	63.7 ± 8.2	1	17	10	162	67	0	29	408	77	N	8
					Control	112	54 (48.2%)	71.0 ± 10.6	4	5	5	78	19	1	18	180	26		

Parfenov et al., 2007 [35]	Yakutsk	CT/MRI	PCR	P-B	Case	107	69 (64.5%)	58.4 ± 11.5	1	5	1	63	33	4	8	164	42	Y	8
					Control	101	61 (59.4%)	57.6 ± 11.6	1	15	3	58	22	2	20	153	29		

Kang and Lee.2006 [36]	Korea	MRI	PCR	H-B	Case	194	116 (59.8%)	62.0 ± 9.5	0	24	0	126	44	0	24	320	44	Y	8
					Control	168	94 (55.9%)	62.3 ± 6.3	2	18	0	128	19	1	22	293	21		
Gao et al., 2006 [37]	China	CT/MRI	PCR	H-B	Case	100	71 (71.0%)	61.1 ± 10.8	1	11	0	75	13	0	13	174	13	Y	8
					Control	100	71 (71.0%)	61.0 ± 10.6	1	13	0	80	6	0	15	179	6		

Baum et al., 2006 [38]	China	CT/MRI	PCR	P-B	Case	243	134 (54.5%)	70.7 ± 12.0	7	39	6	155	32	4	59	381	46	Y	8
					Control	311	152 (45.2%)	70.0 ± 5.9	2	60	6	203	39	1	70	505	47		

Pezzini et al., 2005 [39]	Italy	CT/MRI	PCR	H-B	Case	163	84 (51.5%)	35.0 ± 7.5	2	12	1	109	38	1	17	268	41	Y	8
					Control	158	85 (53.8%)	34.8 ± 6.1	0	16	1	120	21	0	17	277	22		

Cerrato et al., 2005 [40]	Italy	CT/MRI	PCR	P-B	Case	302	100 (33.1%)	57.0 ± 11.0	9	31	0	230	28	4	49	519	36	Y	7
					Control	228	104 (33.1%)	55.0 ± 16.0	3	25	1	158	37	4	32	378			

Jin et al., 2004 [41]	China	CT/MRI	PCR-RFLP	P-B	Case	226	129 (57.1%)	48.5 ± 3.4	2	14	3	152	52	3	21	370	61	Y	8
					Control	201	109 (54.2%)	47.1 ± 2.4	2	17	2	156	22	2	23	351	28		

Duzenli et al., 2004 [42]	Turkey	CT	PCR	P-B	Case	62	NA	NA	0	8	1	52	1	0	9	113	2	Y	8
					Control	126	61 (48.4%)	58.0 ± 1.9	2	23	2	80	18	1	29	201	22		

Slowik et al., 2003 [43]	Poland	CT/MRI	Immuno-blotting	H-B	Case	71	49 (69.0%)	59.6 ± 9.5	0	3	0	53	14	1	3	123	16	Y	7
					Control	30	19 (63.4%)	63.1 ± 8.8	0	1	0	21	8	0	1	51	8		

Souza et al., 2003 [44]	Brazil	CT	PCR	P-B	Case	107	NA	68.8 ± 9.2	0	5	0	93	8	1	5	199	10	Y	8
					Control	100	NA	69.4 ± 8.3	0	8	2	74	16	0	10	172	18		

Karttunen et al., 2002 [45]	Finland	CT/MRI	Immuno-blotting	P-B	Case	44	27 (61.4%)	15–60	0	3	1	27	13	0	4	70	14	Y	8
					Control	104	59 (56.7%)	15–60	1	4	1	67	28	3	7	166	35		

Morrison et al., 2002 [46]	America	MRI	PCR	P-B	Case	400	NA	NA	1	48	19	199	118	15	69	564	167	Y	7
					Control	1104	NA	NA	5	148	39	596	288	28	197	1628	383		
MacLeod et al., 2001 [47]	Scotland	CT	PCR	P-B	Case	266	150 (56.4%)	65.7 ± 12.2	1	29	7	170	56	3	38	425	69	Y	7
					Control	225	94 (41.7%)	77.0 ± 1.0	0	20	6	133	63	3	26	349	75		

Chowdhury et al., 2001 [48]	Bangladesh	CT	PCR	H-B	Case	147	116 (79.9%)	57.9 ± 11.1	3	3	0	113	26	2	9	255	30	N	6
					Control	190	129 (67.7%)	60.3 ± 9.6	3	6	1	149	29	2	13	333	34		

Frikke-Schmidt et al., 2001 [49]	Denmark	CT	PCR	P-B	Case	738	282 (61.8%)	63.0 ± 7.4	5	77	23	409	207	17	110	1102	264	Y	6
					Control	8938	4022 (45.0%)	57.2 ± 0.2	45	1126	232	5050	2244	241	1448	13470	2958		

Catto et al., 2000 [50]	England	CT	PCR	P-B	Case	515	259 (50.3%)	73.0 ± *X*	0	61	8	321	115	10	69	818	143	Y	7
					Control	289	151 (52.2%)	72.5 ± *X*	0	37	7	170	69	6	44	446	88		

Kokubo et al., 2000 [51]	Japan	CT/MRI	PCR-RFLP	P-B	Case	201	NA	40–89	12	15	2	138	33	1	41	324	37	N	7
					Control	1126	333 (29.7%)	64.3 ± 10.5	11	73	8	819	202	13	103	1913	236		

Peng et al., 1999 [52]	China	CT	PCR	H-B	Case	90	NA	62.6 ± 8.9	0	13	1	55	19	2	14	142	24	Y	7
					Control	90	NA	63.1 ± 8.3	1	16	1	63	8	1	19	150	11		

Ji et al., 1998 [53]	Japan	CT/MRI	PCR-RFLP	P-B	Case	123	NA	70.2 ± 7.2	0	9	3	79	29	3	12	196	38	N	7
					Control	117	NA	71.5 ± 7.5	0	4	4	95	14	0	8	208	18		

Margaglione et al., 1998 [54]	Italy	CT/MRI	PCR	P-B	Case	100	51 (51.0%)	66.2 ± 10.0	1	10	0	59	24	6	12	152	36	Y	8
					Control	506	NA	NA	5	47	7	368	78	1	64	861	87		

Kessler et al., 1997 [55]	Germany	CT/MRI	PCR	H-B	Case	227	108 (47.6%)	62.3 ± 14.2	2	31	5	132	50	7	40	345	69	Y	8
					Control	225	108 (48.0%)	62.6 ± 14.0	1	24	6	149	43	2	32	365	53		

Hachinski et al., 1996 [56]	Britain	CT/MRI	IF	P-B	Case	89	61 (67.8%)	64.6 ± 8.7	1	13	1	47	24	3	16	131	31	Y	8
					Control	89	NA	64.5 ± 8.6	2	10	1	57	18	1	15	142	21		
Couderc et al., 1993 [57]	France	CT	IF	H-B	Case	69	36 (52.2%)	72.3 ± 11.6	1	7	0	50	10	1	9	117	12	N	7
					Control	566	347 (61.3%)	41.3 ± 15.3	8	60	5	377	109	7	81	923	128		

Qian et al., 2012 [58]	China	CT/MRI	PCR	H-B	Case	152	87 (57.2%)	66.8 ± 5.5	0	21	0	95	29	7	21	240	43	Y	9
					Control	40	13 (32.5%)	64.0 ± 12.6	0	5	0	29	6	0	5	69	6		

Konialis et al., 2016 [59]	Greece	CT	PCR	H-B	Case	200	142 (72.0%)	60.0 ± 16.0	0	10	3	145	39	3	13	339	48	Y	7
					Control	159	76 (47.5%)	59.0 ± 13.0	1	16	0	126	16	0	18	284	16		

Fayed et al., 2009 [60]	Egypt	CT/MRI	PCR-RFLP	H-B	Case	40	NA	NA	0	3	7	11	11	8	10	36	34	Y	6
					Control	20	NA	NA	0	3	1	15	1	0	4	34	2		

Stankovic et al., 2004 [61]	Serbian	CT/MRI	PCR-RFLP	P-B	Case	65	NA	NA	0	6	0	39	18	2	6	102	22	Y	7
					Control	330	NA	NA	12	56	7	205	47	3	87	513	60		

Pedro-Botet et al., 1992 [62]	Spain	CT	PCR	P-B	Case	100	NA	NA	2	12	0	54	26	6	16	146	38	Y	7
					Control	100	NA	NA	0	13	2	69	13	3	15	164	21		

Fekih-Mrissa et al., 2014 [63]	Tunisia	CT/MRI	PCR	P-B	Case	6	NA	NA	0	0	0	0	5	1	0	5	7	Y	7
					Control	42	NA	NA	0	8	0	18	15	1	8	59	17		

Brewin et al., 2020 [64]	London	CT/MRI	Exome sequenc-ing	P-B	Case	47	NA	NA	0	5	8	14	14	6	13	47	34	Y	7
					Control	236	NA	NA	6	41	11	97	71	10	64	306	102		

Saidi et al., 2009 [65]	Tunisia	CT/MRI	PCR	P-B	Case	228	114 (50.0%)	61.5 ± 12.1	0	14	25	74	87	28	39	249	168	Y	8
					Control	323	177 (54.8%)	60.9 ± 12.8	0	27	28	187	71	10	55	472	119		

Wen et al., 2006 [66]	China	MRI	PCR	P-B	Case	67	NA	70.7 ± 11.4	4	7	2	41	11	2	17	100	17	Y	9
					Control	134	NA	NA	2	24	3	89	15	1	31	217	20		

Giassakis et al., 2007 [67]	Greece	CT/MRI	PCR	P-B	Case	100	70 (70.0%)	60.7 ± 9.8	NA	NA	NA	NA	NA	NA	12	166	22	Y	8
					Control	96	66 (68.8%)	61.3 ± 9.8	NA	NA	NA	NA	NA	NA	10	169	13		
Nakata et al., 1997 [68]	Japan	CT/MRI	PCR	P-B	Case	55	25 (45.0%)	66.0 ± 14.0	NA	NA	NA	NA	NA	NA	2	98	10	Y	7
					Control	61	30 (49.0%)	67.0 ± 8.0	NA	NA	NA	NA	NA	NA	7	110	5		

Szolnoki et al., 2002 [69]	Hungary	MRI	PCR	H-B	Case	689	356 (51.7%)	59.8 ± 17.7	NA	NA	NA	NA	NA	NA	104	934	340	Y	7
					Control	652	341 (52.3%)	59.8 ± 16.9	NA	NA	NA	NA	NA	NA	118	1016	170		

Aalto-Setala et al., 1998 [70]	Finland	CT/MRI	IF	P-B	Case	231	NA	<60	NA	NA	NA	NA	NA	NA	17	350	95	Y	7
					Control	615	NA	20–55	NA	NA	NA	NA	NA	NA	74	861	295		

Artieda et al., 2008 [71]	Spain	CT/MRI	PCR	P-B	Case	152	NA	61.7 ± 6.8	*ε*2/*ε*2 + *ε*2/*ε*3 = 15		1	110	*ε*3/*ε*4 + *ε*4/*ε*4 = 26		NA	NA	NA	Y	7
					Control	215	NA	NA	*ε*2/*ε*2 + *ε*2/*ε*3 = 20		1	164	*ε*3/*ε*4 + *ε*4/*ε*4 = 30		NA	NA	NA		

Schneider et al., 2005 [72]	America	CT	PCR	P-B	Case	76	NA	NA	*ε*2/*ε*2 = 0; *ε*2/*ε*3 + *ε*3/*ε*3 = 45; *ε*2/*ε*4 + *ε*3/*ε*4 + *ε*4/*ε*4 = 31						NA	NA	NA	NA	7
					Control	138	NA	NA	*ε*2/*ε*2 = 0; *ε*2/*ε*3 + *ε*3/*ε*3 = 104; *ε*2/*ε*4 + *ε*3/*ε*4 + *ε*4/*ε*4 = 34						NA	NA	NA		

Li et al., 2016 [73]	China	CT/MRI	PCR	P-B	Case	164	113 (68.9%)	60.8 ± 11.9	*ε*2/*ε*2 + *ε*2/*ε*3 + *ε*3/*ε*3 = 110; *ε*2/*ε*4 + *ε*3/*ε*4 = 42; *ε*4/*ε*4 = 12						NA	NA	NA	Y	8
					Control	109	64 (58.7%)	59.4 ± 13.0	*ε*2/*ε*2 + *ε*2/*ε*3 + *ε*3/*ε*3 = 85; *ε*2/*ε*4 + *ε*3/*ε*4 = 22; *ε*4/*ε*4 = 2						NA	NA	NA		

^∗^Age (years): different statistical patterns of age (mean and IQR, mean ± SD, or range) were extracted. CT: computerized tomography; MRI: magnetic resonance imaging; PCR: polymerase chain reaction; RFLP: restriction fragment length polymorphism; IS: ischemic stroke; *H*: Hardy-Weinberg equilibrium; *N*: Newcastle-Ottawa Scale; NA: not available; IF: isoelectric focusing; H-B: hospital based; P-B: population based.

**Table 2:** The main results of the APOE gene associated with IS included in the meta-analysis.

Genetic model of APOE gene polymorphisms	Group	No. of included studies	Results of association with IS		
			OR	95% CI	*P* value of ORs
*ε*2 allele vs. *ε*3 allele	All	51	0.983	(0.867,1.115)	0.79
	LAA	13	0.962	(0.712,1.299)	0.80
	CE	10	1.517	(0.861,2.674)	0.15
	SAD	12	1.190	(0.997,1.421)	0.05

*ε*4 allele vs. *ε*3 allele	All	51	1.374	(1.214,1.556)	<0.0001
	LAA	13	1.149	(0.898,1.469)	0.27
	CE	10	1.092	(0.662,1.801)	0.73
	SAD	12	1.318	(1.073,1.618)	0.01

*ε*2/*ε*2 vs. *ε*3/3	All	36	0.985	(0.653,1.486)	0.94
	LAA	11	1.307	(0.750,2.278)	0.35
	CE	10	4.290	(1.917,9.600)	<0.0001
	SAD	11	1.803	(1.037,3.134)	0.04

*ε*2/*ε*3 vs. *ε*3/3	All	46	0.980	(0.900,1.066)	0.63
	LAA	13	0.869	(0.705,1.071)	0.19
	CE	10	1.255	(0.849,1.856)	0.26
	SAD	12	1.178	(0.952,1.457)	0.13

*ε*2/*ε*4 vs. *ε*3/3	All	42	1.233	(1.056,1.440)	0.01
	LAA	11	0.978	(0.607,1.576)	0.93
	CE	10	1.458	(0.534,3.980)	0.46
	SAD	10	0.932	(0.526,1.652)	0.81

*ε*3/*ε*4 vs. *ε*3/3	All	47	1.340	(1.165,1.542)	<0.0001
	LAA	14	1.154	(0.841,1.584)	0.38
	CE	10	1.175	(0.627,2.203)	0.62
	SAD	13	1.392	(1.097,1.767)	0.01

*ε*4/*ε*4 vs. *ε*3/3	All	46	1.833	(1.542,2.179)	<0.0001
	LAA	13	1.367	(0.836,2.236)	0.21
	CE	10	1.543	(0.591,4.029)	0.38
	SAD	11	1.809	(1.030,3.175)	0.04

*ε*4 vs. non-*ε*4	All	50	1.377	(1.203,1.576)	<0.0001
	LAA	14	1.149	(0.876,1.506)	0.32
	CE	10	1.091	(0.645,1.845)	0.74
	SAD	13	1.329	(1.064,1.661)	0.01

*ε*2 vs. non-*ε*2	All	48	0.956	(0.841,1.086)	0.49
	LAA	14	0.861	(0.717,1.035)	0.11
	CE	10	1.358	(0.966,1.910)	0.08
	SAD	13	1.117	(0.926,1.347)	0.25

*ε*4/*ε*4 vs. *ε*2/4	All	40	1.625	(1.281,2.060)	<0.0001
	LAA	11	1.551	(0.791,3.043)	0.20
	CE	9	0.771	(0.177,3.352)	0.73
	SAD	4	2.115	(0.919,4.867)	0.08

*ε*4/*ε*4 vs. *ε*3/4	All	46	1.301	(1.077,1.571)	0.01
	LAA	13	1.353	(0.811,2.258)	0.25
	CE	6	1.077	(0.402,2.887)	0.88
	SAD	11	1.332	(0.739,2.400)	0.34

## Data Availability

Data presented within the paper and the supplementary materials contributed to the findings in our study. They are all are available from our corresponding author for reasonable request.

## Supplementary Materials

Supplementary Materials Supplementary material Table 1: fifteen of the included studies provide data about different subtypes of IS: LAA, SAD, and CE. Supplementary material Table 2: Newcastle-Ottawa Scale (NOS) score of included studies. Supplementary material Table 3: PRISMA list of our meta-analysis. Supplementary material Table 4: sensitivity analysis of the association between ApoE gene polymorphisms and IS. Supplementary material Table 5: publication bias and heterogeneity of our meta-analysis. Supplementary material Figure 1: funnel plots for studies included in Figures 2A–G. Supplementary material Figure 2: funnel plots for studies included in Figures 3A–G. Supplementary material Figure 3: results of meta-regression.
