# Supplementary material for: Apolipoprotein E ε4 Polymorphism as a Risk Factor for Ischemic Stroke: A Systematic Review and Meta-Analysis
Source: Dis Markers. 2022 Feb 3;2022:1407183. doi: 10.1155/2022/1407183 (PMC8831053; doi:10.1155/2022/1407183)
Supplement: Supplementary Materials — Supplementary material Table 1: fifteen of the included studies provide data about different subtypes of IS: LAA, SAD, and CE. Supplementary material Table 2: Newcastle-Ottawa Scale (NOS) score of included studies. Supplementary material Table 3: PRISMA list of our meta-analysis. Supplementary material Table 4: sensitivity analysis of the association between ApoE gene polymorphisms and IS. Supplementary material Table 5: publication bias and heterogeneity of our meta-analysis. Supplementary material Figure 1: funnel plots for studies included in Figures 2A–G. Supplementary material Figure 2: funnel plots for studies included in Figures 3A–G. Supplementary material Figure 3: results of meta-regression. [file 1407183.f1.zip › Supplementary materials.Figure 2.pdf]

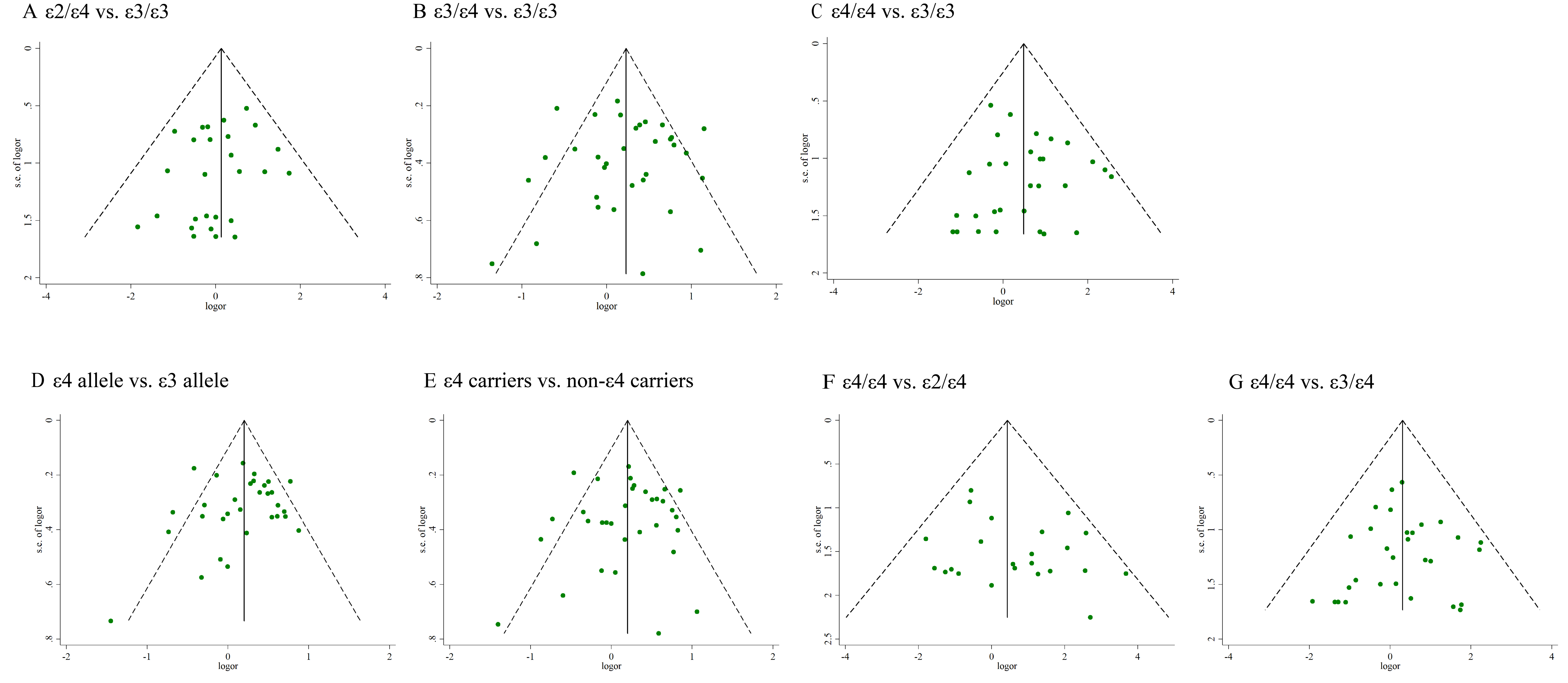
A ε2/ε4 vs. ε3/ε3
B ε3/ε4 vs. ε3/ε3
C ε4/ε4 vs. ε3/ε3
D ε4 allele vs. ε3 allele
E ε4 carriers vs. non-ε4 carriers
F ε4/ε4 vs. ε2/ε4
G ε4/ε4 vs. ε3/ε4
s.c. of logor
logor
