# Supplementary material for: Apolipoprotein E ε4 Polymorphism as a Risk Factor for Ischemic Stroke: A Systematic Review and Meta-Analysis
Source: Dis Markers. 2022 Feb 3;2022:1407183. doi: 10.1155/2022/1407183 (PMC8831053; doi:10.1155/2022/1407183)
Supplement: Supplementary Materials — Supplementary material Table 1: fifteen of the included studies provide data about different subtypes of IS: LAA, SAD, and CE. Supplementary material Table 2: Newcastle-Ottawa Scale (NOS) score of included studies. Supplementary material Table 3: PRISMA list of our meta-analysis. Supplementary material Table 4: sensitivity analysis of the association between ApoE gene polymorphisms and IS. Supplementary material Table 5: publication bias and heterogeneity of our meta-analysis. Supplementary material Figure 1: funnel plots for studies included in Figures 2A–G. Supplementary material Figure 2: funnel plots for studies included in Figures 3A–G. Supplementary material Figure 3: results of meta-regression. [file 1407183.f1.zip › Supplementary materials.Figure 3.pdf]

A ε2 carriers vs. non ε2 carriers

```
Meta-regression                                       Number of obs  =        47
REML estimate of between-study variance               tau2           =     .1026
% residual variation due to heterogeneity             I-squared_res  =    55.99%
Proportion of between-study variance explained        Adj R-squared  =    -7.00%
Joint test for all covariates                         Model F(7,39)  =      0.63
With Knapp-Hartung modification                       Prob > F       =    0.7305
```

logor	exp(b)	Std. Err.	t	P>|t|	[95% Conf.	Interval]
region	.8623136	.1359714	-0.94	0.353	.6268309	1.18626
sample_size	1.051511	.1889355	0.28	0.781	.731097	1.512351
genotyping_method	.9705757	.2554884	-0.11	0.910	.5698931	1.652972
hwe	.7928457	.1470492	-1.25	0.218	.5448333	1.153755
year	1.003297	.0115209	0.29	0.776	.9802626	1.026873
nos	1.323399	.3404652	1.09	0.283	.7864976	2.226815
source	.9720725	.1452431	-0.19	0.851	.7185305	1.31508
_cons	.0014338	.0330504	-0.28	0.778	8.07e-24	2.55e+17

B ε4 carriers vs. non ε4 carriers

```
Meta-regression                                       Number of obs  =        50
REML estimate of between-study variance               tau2           =     .1721
% residual variation due to heterogeneity             I-squared_res  =    73.66%
Proportion of between-study variance explained        Adj R-squared  =    -5.02%
Joint test for all covariates                         Model F(7,42)  =      0.68
With Knapp-Hartung modification                       Prob > F       =    0.6850
```

logor	exp(b)	Std. Err.	t	P>|t|	[95% Conf.	Interval]
region	.9249501	.1757586	-0.41	0.683	.6303434	1.357248
sample_size	.8699413	.1975984	-0.61	0.543	.5500663	1.37583
genotyping_method	1.291803	.3864977	0.86	0.397	.7062716	2.362768
hwe	.8480877	.1989668	-0.70	0.486	.5282286	1.361632
year	1.015473	.0135435	1.15	0.256	.9885054	1.043176
nos	.7901216	.2429099	-0.77	0.448	.4248605	1.469405
source	.8321578	.1480539	-1.03	0.308	.5811296	1.191622
_cons	6.75e-14	1.81e-12	-1.13	0.264	2.28e-37	2.00e+10

C ε2 alleles vs. ε3 alleles

```
Meta-regression                                       Number of obs  =        51
REML estimate of between-study variance               tau2           =     .1002
% residual variation due to heterogeneity             I-squared_res  =    60.02%
Proportion of between-study variance explained        Adj R-squared  =     1.56%
Joint test for all covariates                         Model F(7,43)  =      0.72
With Knapp-Hartung modification                       Prob > F       =    0.6543
```

logor	exp(b)	Std. Err.	t	P>|t|	[95% Conf.	Interval]
region	.9239362	.12843	-0.57	0.572	.698068	1.222887
sample_size	.8473395	.1390246	-1.01	0.318	.6086351	1.179663
genotyping_method	.9922835	.2775972	-0.03	0.978	.5644361	1.744443
hwe	1.368335	.2535057	1.69	0.098	.9417372	1.988178
year	1.009614	.0107363	0.90	0.373	.9881927	1.0315
nos	.8333639	.2523	-0.60	0.550	.4525595	1.534595
source	.9111102	.1250359	-0.68	0.501	.6908368	1.201618
_cons	4.86e-09	1.04e-07	-0.90	0.375	9.92e-28	2.38e+10

D ε4 alleles vs. ε3 alleles

```
Meta-regression                                       Number of obs  =        51
REML estimate of between-study variance               tau2           =     .1439
% residual variation due to heterogeneity             I-squared_res  =    75.41%
Proportion of between-study variance explained        Adj R-squared  =    -3.33%
Joint test for all covariates                         Model F(7,43)  =      0.97
With Knapp-Hartung modification                       Prob > F       =    0.4673
```

logor	exp(b)	Std. Err.	t	P>|t|	[95% Conf.	Interval]
region	1.060643	.1845909	0.34	0.737	.7466912	1.506598
sample_size	.8575302	.1478732	-0.89	0.378	.6056464	1.214171
genotyping_method	.6232819	.1726529	-1.71	0.095	.3565102	1.089675
hwe	.9001164	.1899843	-0.50	0.621	.5880831	1.377713
year	.9994372	.0117455	-0.05	0.962	.9760285	1.023407
nos	.9759355	.2847505	-0.08	0.934	.5418464	1.757786
source	1.303242	.211836	1.63	0.111	.9389914	1.808792
_cons	4.075754	96.15793	0.06	0.953	8.85e-21	1.88e+21

E ε3/ε4 vs. ε3/ε3

```
Meta-regression                                       Number of obs  =        45
REML estimate of between-study variance               tau2           =      .113
% residual variation due to heterogeneity             I-squared_res  =    60.26%
Proportion of between-study variance explained        Adj R-squared  =    29.31%
Joint test for all covariates                         Model F(7,37)  =      1.46
With Knapp-Hartung modification                       Prob > F       =    0.2127
```

logor	exp(b)	Std. Err.	t	P>|t|	[95% Conf.	Interval]
region	.7422095	.129962	-1.70	0.097	.5205272	1.058302
sample_size	1.000142	.2103739	0.00	0.999	.6530802	1.531641
genotyping_method	.7612175	.2375813	-0.87	0.388	.404451	1.432688
hwe	.9740102	.2218622	-0.12	0.909	.6139363	1.545268
year	1.004617	.0135686	0.34	0.735	.9774967	1.032489
nos	1.07659	.3698069	0.21	0.831	.5367645	2.159318
source	1.25893	.2210248	1.31	0.198	.8820848	1.796773
_cons	.0001402	.0037985	-0.33	0.745	2.05e-28	9.61e+19
