# Supplementary material for: Apolipoprotein E ε4 Polymorphism as a Risk Factor for Ischemic Stroke: A Systematic Review and Meta-Analysis
Source: Dis Markers. 2022 Feb 3;2022:1407183. doi: 10.1155/2022/1407183 (PMC8831053; doi:10.1155/2022/1407183)
Supplement: Supplementary Materials — Supplementary material Table 1: fifteen of the included studies provide data about different subtypes of IS: LAA, SAD, and CE. Supplementary material Table 2: Newcastle-Ottawa Scale (NOS) score of included studies. Supplementary material Table 3: PRISMA list of our meta-analysis. Supplementary material Table 4: sensitivity analysis of the association between ApoE gene polymorphisms and IS. Supplementary material Table 5: publication bias and heterogeneity of our meta-analysis. Supplementary material Figure 1: funnel plots for studies included in Figures 2A–G. Supplementary material Figure 2: funnel plots for studies included in Figures 3A–G. Supplementary material Figure 3: results of meta-regression. [file 1407183.f1.zip › Supplementary tables.docx]

**Supplementary material Table 1. Fifteen of the included studies provide data about different subtypes of IS: LAA, SAD, CE.**

| Study ID | Type | Case group | | | | | |  |  |  | Control group | | | | | |  |  |  |
| --- | --- | --- | --- | --- | --- | --- | --- | --- | --- | --- | --- | --- | --- | --- | --- | --- | --- | --- | --- |
|  | | ε2/ε2 | ε2/ε3 | ε2/ε4 | ε3/ε3 | ε3/ε4 | ε4/ε4 | ε2 | ε3 | ε4 | ε2/ε2 | ε2/ε3 | ε2/ε4 | ε3/ε3 | ε3/ε4 | ε4/ε4 | ε2 | ε3 | ε4 |
| Zhao et al.2017 | LAA | 0 | 10 | 0 | 85 | 21 | 3 | 10 | 201 | 27 | 5 | 70 | 8 | 366 | 64 | 1 | 88 | 866 | 74 |
|  | CE | 1 | 1 | 0 | 21 | 4 | 0 | 3 | 47 | 4 | 5 | 70 | 8 | 366 | 64 | 1 | 88 | 866 | 74 |
|  | SAD | 2 | 33 | 3 | 165 | 34 | 5 | 40 | 397 | 47 | 5 | 70 | 8 | 366 | 64 | 1 | 88 | 866 | 74 |
| Das et al.2016 | LAA | 2 | 15 | 3 | 243 | 35 | 5 | 22 | 536 | 48 | 5 | 50 | 4 | 436 | 113 | 12 | 64 | 1035 | 141 |
|  | CE | 2 | 12 | 0 | 33 | 27 | 2 | 16 | 105 | 31 | 5 | 50 | 4 | 436 | 113 | 12 | 64 | 1035 | 141 |
|  | SAD | 1 | 4 | 2 | 50 | 25 | 1 | 8 | 129 | 29 | 5 | 50 | 4 | 436 | 113 | 12 | 64 | 1035 | 141 |
| Luo et al.2015 | LAA | 2 | 35 | 7 | 225 | 54 | 4 | 46 | 539 | 69 | 3 | 107 | 8 | 535 | 113 | 8 | 121 | 1290 | 137 |
|  | CE | 1 | 6 | 1 | 38 | 8 | 0 | 9 | 90 | 9 | 3 | 107 | 8 | 535 | 113 | 8 | 121 | 1290 | 137 |
|  | SAD | 1 | 36 | 2 | 152 | 28 | 2 | 40 | 368 | 34 | 3 | 107 | 8 | 535 | 113 | 8 | 121 | 1290 | 137 |
| Chatzistefanidis et al.2014 | LAA | 1 | 14 | 1 | 108 | 27 | 2 | 17 | 257 | 32 | 2 | 24 | 8 | 278 | 47 | 2 | 36 | 627 | 59 |
|  | SAD | 2 | 23 | 2 | 116 | 31 | 2 | 29 | 286 | 37 | 2 | 24 | 8 | 278 | 47 | 2 | 36 | 627 | 59 |
| Tascilar et al.2009 | LAA | 3 | 18 | 3 | 45 | 9 | 7 | 27 | 117 | 26 | 3 | 16 | 7 | 40 | 9 | 2 | 29 | 105 | 20 |
| Lai et al.2007 | LAA | 1 | 7 | 6 | 77 | 23 | 0 | 15 | 184 | 29 | 4 | 5 | 5 | 78 | 19 | 1 | 18 | 180 | 26 |
|  | SAD | 0 | 10 | 4 | 85 | 44 | 0 | 14 | 224 | 48 | 4 | 5 | 5 | 78 | 19 | 1 | 18 | 180 | 26 |
| Kang and Lee.2006 | LAA | 0 | 11 | 0 | 76 | 25 | 0 | 11 | 188 | 25 | 2 | 18 | 0 | 128 | 19 | 1 | 22 | 293 | 21 |
|  | SAD | 0 | 13 | 0 | 50 | 19 | 0 | 13 | 132 | 19 | 2 | 18 | 0 | 128 | 19 | 1 | 22 | 293 | 21 |
| Gao et al.2006 | LAA | ε2/ε2＋ε2/ε3=1 | | 0 | 25 | 5 | 0 | NA | NA | 5 | ε2/ε2＋ε2/ε3=5 | | 0 | 23 | 3 | 0 | NA | NA | 3 |
|  | SAD | ε2/ε2＋ε2/ε3=11 | | 0 | 50 | 8 | 0 | NA | NA | 8 | ε2/ε2＋ε2/ε3=9 | | 0 | 57 | 3 | 0 | NA | NA | 3 |
| Cerrato et al.2005 | LAA | 4 | 9 | 0 | 88 | 10 | 1 | 17 | 195 | 12 | 3 | 25 | 1 | 158 | 37 | 4 | 32 | 378 | 46 |
|  | CE | 1 | 2 | 0 | 33 | 2 | 0 | 4 | 70 | 2 | 3 | 25 | 1 | 158 | 37 | 4 | 32 | 378 | 46 |
|  | SAD | 4 | 9 | 0 | 52 | 11 | 0 | 17 | 124 | 11 | 3 | 25 | 1 | 158 | 37 | 4 | 32 | 378 | 46 |
| Slowik et al.2003 | LAA | 0 | 1 | 0 | 24 | 4 | 1 | 1 | 53 | 6 | 0 | 1 | 0 | 21 | 8 | 0 | 1 | 51 | 8 |
|  | SAD | 0 | 2 | 0 | 29 | 10 | 0 | 2 | 70 | 10 | 0 | 1 | 0 | 21 | 8 | 0 | 1 | 51 | 8 |
| Souza et al.2003 | LAA | 0 | 5 | 0 | 93 | 8 | 1 | 5 | 199 | 10 | 0 | 8 | 2 | 74 | 16 | 0 | 10 | 172 | 18 |
| Kokubo et al.2000 | LAA | 4 | 8 | 1 | 32 | 17 | 0 | 17 | 89 | 18 | 11 | 73 | 8 | 819 | 202 | 13 | 103 | 1913 | 236 |
|  | CE | 3 | 3 | 1 | 18 | 6 | 0 | 10 | 45 | 7 | 11 | 73 | 8 | 819 | 202 | 13 | 103 | 1913 | 236 |
|  | SAD | 2 | 2 | 0 | 59 | 10 | 1 | 6 | 130 | 12 | 11 | 73 | 8 | 819 | 202 | 13 | 103 | 1913 | 236 |
| Kessler et al.1997 | LAA | 0 | 6 | 4 | 39 | 20 | 1 | 10 | 104 | 26 | 1 | 24 | 6 | 149 | 43 | 2 | 32 | 365 | 53 |
|  | CE | 0 | 10 | 1 | 32 | 9 | 1 | 11 | 83 | 12 | 1 | 24 | 6 | 149 | 43 | 2 | 32 | 365 | 53 |
|  | SAD | 2 | 4 | 0 | 18 | 8 | 2 | 8 | 48 | 12 | 1 | 24 | 6 | 149 | 43 | 2 | 32 | 365 | 53 |
| Pedro-Botet et al.1992 | LAA | 1 | 7 | 0 | 24 | 14 | 2 | 9 | 69 | 18 | 0 | 13 | 2 | 69 | 13 | 3 | 15 | 164 | 21 |
|  | SAD | 1 | 3 | 0 | 15 | 6 | 3 | 5 | 39 | 12 | 0 | 13 | 2 | 69 | 13 | 3 | 15 | 164 | 21 |
| Wen et al.2006 | SAD | 4 | 7 | 2 | 41 | 11 | 2 | 17 | 100 | 17 | 2 | 24 | 3 | 89 | 15 | 1 | 31 | 217 | 20 |

**Supplementary material Table 2. Newcastle-Ottawa Scale (NOS) score of included studies.**

| Study ID | Selection | | | |  | Comparability | |  | Exposure | | | NOS score |
| --- | --- | --- | --- | --- | --- | --- | --- | --- | --- | --- | --- | --- |
|  | 1 | 2 | 3 | 4 |  | 5a | 5b |  | 6 | 7 | 8 |  |
| Wu et al.2020 | Yes | Yes | Yes | Yes |  | No | Yes |  | Yes | Yes | Yes | 8 |
| Zhao et al.2017 | Yes | Yes | Yes | Yes |  | No | No |  | Yes | Yes | Yes | 7 |
| Coen Herak et al.2017 | Yes | Yes | Yes | Yes |  | Yes | No |  | Yes | Yes | Yes | 8 |
| Das et al.2016 | Yes | Yes | Yes | Yes |  | No | Yes |  | Yes | Yes | Yes | 8 |
| Koopal et al.2016 | Yes | Yes | Yes | Yes |  | No | No |  | Yes | Yes | Yes | 7 |
| Luo et al.2015 | Yes | Yes | Yes | Yes |  | No | No |  | Yes | Yes | Yes | 7 |
| Wei et al.2015 | Yes | Yes | Yes | Yes |  | No | Yes |  | Yes | Yes | Yes | 8 |
| Yan et al.2015 | Yes | Yes | Yes | Yes |  | No | Yes |  | Yes | Yes | Yes | 8 |
| Chatzistefanidis et al.2014 | Yes | Yes | No | Yes |  | No | Yes |  | Yes | Yes | Yes | 7 |
| Atadzhanov et al.2013 | Yes | Yes | Yes | Yes |  | Yes | Yes |  | Yes | Yes | Yes | 9 |
| Gelfand et al.2013 | Yes | Yes | Yes | Yes |  | No | Yes |  | Yes | Yes | Yes | 8 |
| Balcerzyk et al.2010 | Yes | Yes | Yes | Yes |  | No | No |  | Yes | Yes | Yes | 7 |
| Tamam et al.2009 | Yes | Yes | Yes | Yes |  | No | No |  | Yes | Yes | Yes | 7 |
| Tascilar et al.2009 | Yes | Yes | Yes | Yes |  | No | No |  | Yes | Yes | Yes | 7 |
| Wang et al.2009 | Yes | Yes | No | Yes |  | No | Yes |  | Yes | Yes | Yes | 7 |
| Lai et al.2007 | Yes | Yes | Yes | Yes |  | No | Yes |  | Yes | Yes | Yes | 8 |
| Parfenov et al.2007 | Yes | Yes | Yes | Yes |  | No | Yes |  | Yes | Yes | Yes | 8 |
| Kang and Lee.2006 | Yes | Yes | No | Yes |  | Yes | Yes |  | Yes | Yes | Yes | 8 |
| Gao et al.2006 | Yes | Yes | No | Yes |  | Yes | Yes |  | Yes | Yes | Yes | 8 |
| Baum et al.2006 | Yes | Yes | Yes | Yes |  | Yes | No |  | Yes | Yes | Yes | 8 |
| Pezzini et al.2005 | Yes | Yes | Yes | Yes |  | No | Yes |  | Yes | Yes | Yes | 8 |
| Cerrato et al.2005 | Yes | Yes | Yes | Yes |  | No | No |  | Yes | Yes | Yes | 7 |
| Jin et al.2004 | Yes | Yes | Yes | Yes |  | No | Yes |  | Yes | Yes | Yes | 8 |
| Duzenli et al.2004 | Yes | Yes | Yes | Yes |  | No | Yes |  | Yes | Yes | Yes | 8 |
| Slowik et al.2003 | Yes | Yes | No | Yes |  | No | Yes |  | Yes | Yes | Yes | 7 |
| Souza et al.2003 | Yes | Yes | Yes | Yes |  | No | Yes |  | Yes | Yes | Yes | 8 |
| Karttunen et al.2002 | Yes | Yes | Yes | Yes |  | No | Yes |  | Yes | Yes | Yes | 8 |
| Morrison et al.2002 | Yes | Yes | Yes | Yes |  | No | No |  | Yes | Yes | Yes | 7 |
| MacLeod et al.2001 | Yes | Yes | Yes | Yes |  | No | No |  | Yes | Yes | Yes | 7 |
| Chowdhury et al.2001 | Yes | Yes | No | Yes |  | No | No |  | Yes | Yes | Yes | 6 |
| Frikke-Schmidt et al.2001 | Yes | Yes | Yes | Yes |  | No | No |  | Yes | Yes | Yes | 7 |
| Catto et al.2000 | Yes | Yes | Yes | Yes |  | No | No |  | Yes | Yes | Yes | 7 |
| Kokubo et al.2000 | Yes | Yes | Yes | Yes |  | No | No |  | Yes | Yes | Yes | 7 |
| Peng et al.1999 | Yes | Yes | No | Yes |  | No | Yes |  | Yes | Yes | Yes | 7 |
| Ji et al.1998 | Yes | Yes | No | Yes |  | No | Yes |  | Yes | Yes | Yes | 7 |
| Margaglione et al.1998 | Yes | Yes | No | Yes |  | Yes | Yes |  | Yes | Yes | Yes | 8 |
| Kessler et al.1997 | Yes | Yes | Yes | Yes |  | No | Yes |  | Yes | Yes | Yes | 8 |
| Hachinski et al.1996 | Yes | Yes | Yes | Yes |  | No | Yes |  | Yes | Yes | Yes | 8 |
| Couderc et al. 1993 | Yes | Yes | Yes | Yes |  | No | No |  | Yes | Yes | Yes | 7 |
| Qian et al.2012 | Yes | Yes | Yes | Yes |  | Yes | Yes |  | Yes | Yes | Yes | 9 |
| Konialis et al.2016 | Yes | Yes | Yes | Yes |  | No | No |  | Yes | Yes | Yes | 7 |
| Fayed et al.2009 | Yes | Yes | No | Yes |  | No | No |  | Yes | Yes | Yes | 6 |
| Stankovic et al.2004 | Yes | Yes | Yes | Yes |  | No | No |  | Yes | Yes | Yes | 7 |
| Pedro-Botet et al.1992 | Yes | Yes | Yes | Yes |  | No | No |  | Yes | Yes | Yes | 7 |
| Fekih-Mrissa et al.2014 | Yes | Yes | Yes | Yes |  | No | No |  | Yes | Yes | Yes | 7 |
| Brewin et al.2020 | Yes | Yes | Yes | Yes |  | No | No |  | Yes | Yes | Yes | 7 |
| Saidi et al.2009 | Yes | Yes | Yes | Yes |  | No | Yes |  | Yes | Yes | Yes | 8 |
| Wen et al.2006 | Yes | Yes | Yes | Yes |  | Yes | Yes |  | Yes | Yes | Yes | 9 |
| Giassakis et al.2007 | Yes | Yes | Yes | Yes |  | No | Yes |  | Yes | Yes | Yes | 8 |
| Nakata et al.1997 | Yes | Yes | Yes | Yes |  | No | No |  | Yes | Yes | Yes | 7 |
| Szolnoki et al.2002 | Yes | Yes | No | Yes |  | No | Yes |  | Yes | Yes | Yes | 7 |
| Aalto-Setala et al.1998 | Yes | Yes | Yes | Yes |  | No | No |  | Yes | Yes | Yes | 7 |
| Artieda et al.2008 | Yes | Yes | Yes | Yes |  | No | No |  | Yes | Yes | Yes | 7 |
| Schneider et al.2005 | Yes | Yes | Yes | Yes |  | No | No |  | Yes | Yes | Yes | 7 |
| Li et al. 2016 | Yes | Yes | Yes | Yes |  | No | Yes |  | Yes | Yes | Yes | 8 |

**Supplementary material Table 3.** **PRISMA list of our Meta-analysis.**

| **Section and Topic** | **Item #** | **Checklist item** | **Location where item is reported** |
| --- | --- | --- | --- |
| **TITLE** | | |  |
| Title | 1 | Identify the report as a systematic review. | Page1, line1-3 |
| **ABSTRACT** | | |  |
| Abstract | 2 | See the PRISMA 2020 for Abstracts checklist. | Page2, line1-21 |
| **INTRODUCTION** | | |  |
| Rationale | 3 | Describe the rationale for the review in the context of existing knowledge. | Page3, line2-19 |
| Objectives | 4 | Provide an explicit statement of the objective(s) or question(s) the review addresses. | Page3, line19-22 |
| **METHODS** | | |  |
| Eligibility criteria | 5 | Specify the inclusion and exclusion criteria for the review and how studies were grouped for the syntheses. | Page4, line9-16 |
| Information sources | 6 | Specify all databases, registers, websites, organisations, reference lists and other sources searched or consulted to identify studies. Specify the date when each source was last searched or consulted. | Page4, line1-3 |
| Search strategy | 7 | Present the full search strategies for all databases, registers and websites, including any filters and limits used. | Page4, line3-7 |
| Selection process | 8 | Specify the methods used to decide whether a study met the inclusion criteria of the review, including how many reviewers screened each record and each report retrieved, whether they worked independently, and if applicable, details of automation tools used in the process. | Page4, line9-10 |
| Data collection process | 9 | Specify the methods used to collect data from reports, including how many reviewers collected data from each report, whether they worked independently, any processes for obtaining or confirming data from study investigators, and if applicable, details of automation tools used in the process. | Page4, line18-19 |
| Data items | 10a | List and define all outcomes for which data were sought. Specify whether all results that were compatible with each outcome domain in each study were sought (e.g. for all measures, time points, analyses), and if not, the methods used to decide which results to collect. | Page4, line19-24 |
|  | 10b | List and define all other variables for which data were sought (e.g. participant and intervention characteristics, funding sources). Describe any assumptions made about any missing or unclear information. | Page4, line19-24 |
| Study risk of bias assessment | 11 | Specify the methods used to assess risk of bias in the included studies, including details of the tool(s) used, how many reviewers assessed each study and whether they worked independently, and if applicable, details of automation tools used in the process. | Page4, line26-27 |
| Effect measures | 12 | Specify for each outcome the effect measure(s) (e.g. risk ratio, mean difference) used in the synthesis or presentation of results. | Page5, line3 |
| Synthesis methods | 13a | Describe the processes used to decide which studies were eligible for each synthesis (e.g. tabulating the study intervention characteristics and comparing against the planned groups for each synthesis (item #5)). | Page4, line10-12 |
|  | 13b | Describe any methods required to prepare the data for presentation or synthesis, such as handling of missing summary statistics, or data conversions. | Page5, line3-11 |
|  | 13c | Describe any methods used to tabulate or visually display results of individual studies and syntheses. | Page5, line3-11 |
|  | 13d | Describe any methods used to synthesize results and provide a rationale for the choice(s). If meta-analysis was performed, describe the model(s), method(s) to identify the presence and extent of statistical heterogeneity, and software package(s) used. | Page5, line1-19 |
|  | 13e | Describe any methods used to explore possible causes of heterogeneity among study results (e.g. subgroup analysis, meta-regression). | Page5, line13-19 |
|  | 13f | Describe any sensitivity analyses conducted to assess robustness of the synthesized results. | Page5, line15-16 |
| Reporting bias assessment | 14 | Describe any methods used to assess risk of bias due to missing results in a synthesis (arising from reporting biases). | Page5, line17 |
| Certainty assessment | 15 | Describe any methods used to assess certainty (or confidence) in the body of evidence for an outcome. | Page5, line20-29 |
| **RESULTS** | | |  |
| Study selection | 16a | Describe the results of the search and selection process, from the number of records identified in the search to the number of studies included in the review, ideally using a flow diagram. | Page7, Figure1 |
|  | 16b | Cite studies that might appear to meet the inclusion criteria, but which were excluded, and explain why they were excluded. | Page6, line5-6 |
| Study characteristics | 17 | Cite each included study and present its characteristics. | Page6, line9-17 |
| Risk of bias in studies | 18 | Present assessments of risk of bias for each included study. | Page6, line17-18 |
| Results of individual studies | 19 | For all outcomes, present, for each study: (a) summary statistics for each group (where appropriate) and (b) an effect estimate and its precision (e.g. confidence/credible interval), ideally using structured tables or plots. | Page8-15; Page17-18 |
| Results of syntheses | 20a | For each synthesis, briefly summarise the characteristics and risk of bias among contributing studies. | Page22, line13-18 |
|  | 20b | Present results of all statistical syntheses conducted. If meta-analysis was done, present for each the summary estimate and its precision (e.g. confidence/credible interval) and measures of statistical heterogeneity. If comparing groups, describe the direction of the effect. | Page17-18 |
|  | 20c | Present results of all investigations of possible causes of heterogeneity among study results. | Page22, line13-18 |
|  | 20d | Present results of all sensitivity analyses conducted to assess the robustness of the synthesized results. | Page22, line2-4 |
| Reporting biases | 21 | Present assessments of risk of bias due to missing results (arising from reporting biases) for each synthesis assessed. | Page22, line6-10 |
| Certainty of evidence | 22 | Present assessments of certainty (or confidence) in the body of evidence for each outcome assessed. | Page22, line20-23 |
| **DISCUSSION** | | |  |
| Discussion | 23a | Provide a general interpretation of the results in the context of other evidence. | Page24, line23-32 |
|  | 23b | Discuss any limitations of the evidence included in the review. | Page25, line9-13 |
|  | 23c | Discuss any limitations of the review processes used. | Page25, line30-40; Page26, line1 |
|  | 23d | Discuss implications of the results for practice, policy, and future research. | Page26, line4-7 |
| **OTHER INFORMATION** | | |  |
| Registration and protocol | 24a | Provide registration information for the review, including register name and registration number, or state that the review was not registered. | Unregistered |
|  | 24b | Indicate where the review protocol can be accessed, or state that a protocol was not prepared. | Unregistered |
|  | 24c | Describe and explain any amendments to information provided at registration or in the protocol. | Unregistered |
| Support | 25 | Describe sources of financial or non-financial support for the review, and the role of the funders or sponsors in the review. | Page26, line14 |
| Competing interests | 26 | Declare any competing interests of review authors. | Page26, line16 |
| Availability of data, code and other materials | 27 | Report which of the following are publicly available and where they can be found: template data collection forms; data extracted from included studies; data used for all analyses; analytic code; any other materials used in the review. | Page26, line20-21 |

**Supplementary material Table 4. Sensitivity analysis of association between ApoE Gene Polymorphisms and IS.**

| Study omitted | ε2 allele vs ε3 allele | ε4 allele vs ε3 allele | ε2/ε2 vs ε3/ε3 | ε2/ε3 vs ε3/ε3 | ε2/ε4 vs ε3/ε3 | ε3/ε4 vs ε3/ε3 | ε4/ε4 vs ε3/ε3 | ε4 carriers vs non ε4 carriers | ε2 carriers vs non ε2 carriers | ε4/ε4 vs ε2/4 | ε4/ε4 vs ε3/4 |
| --- | --- | --- | --- | --- | --- | --- | --- | --- | --- | --- | --- |
| Wu et al.2020 | 1.000(0.883,1.134) | 1.366(1.203,1.551) | 1.037(0.684,1.572) | 1.029(0.943,1.123) | 1.219(1.040,1.429) | 1.331(1.152,1.538) | 1.803(1.512,2.150) | 1.366(1.190,1.568) | 0.977(0.862,1.107) | 1.618(1.269,2.061) | 1.286(1.061,1.560) |
| Zhao et al.2017 | 0.986(0.866,1.124) | 1.372(1.208,1.558) | 1.002(0.655,1.533) | 0.981(0.900,1.070) | 1.242(1.061,1.453) | 1.339(1.158,1.547) | 1.798(1.511,2.140) | 1.375(1.197,1.580) | 0.960(0.841,1.095) | 1.584(1.247,2.012) | 1.274(1.053,1.541) |
| Coen Herak etal.2017 | 0.979(0.862,1.112) | 1.377(1.215,1.560) | 0.994(0.655,1.509) | 0.977(0.898,1.064) | 1.225(1.048,1.431) | 1.342(1.164,1.546) | 1.842(1.549,2.191) | 1.378(1.202,1.580) | 0.950(0.835,1.081) | 1.647(1.298,2.090) | 1.307(1.082,1.580) |
| Das et al.2016 | 0.983(0.864,1.120) | 1.385(1.219,1.573) | 0.983(0.641,1.509) | 0.982(0.901,1.070) | 1.229(1.051,1.437) | 1.350(1.168,1.562) | 1.889(1.583,2.255) | 1.387(1.207,1.595) | 0.956(0.838,1.091) | 1.664(1.308,2.117) | 1.324(1.091,1.607) |
| Koopal et al.2016 | 0.979(0.859,1.114) | 1.390(1.225,1.576) | 0.988(0.643,1.518) | 0.972(0.892,1.060) | 1.232(1.051,1.444) | 1.357(1.175,1.567) | 1.924(1.607,2.303) | 1.394(1.214,1.599) | 0.949(0.832,1.082) | 1.706(1.336,2.179) | 1.329(1.093,1.616) |
| Luo et al.2015 | 0.982(0.861,1.119) | 1.387(1.221,1.574) | 0.971(0.635,1.483) | 0.983(0.903,1.073) | 1.219(1.041,1.430) | 1.355(1.173,1.565) | 1.872(1.571,2.231) | 1.390(1.210,1.597) | 0.954(0.835,1.090) | 1.686(1.324,2.148) | 1.314(1.084,1.592) |
| Wei et al.2015 | 0.956(0.849,1.075) | 1.393(1.230,1.578) | 0.947(0.621,1.442） | 0.934(0.857,1.017) | 1.251(1.065,1.469) | 1.365(1.186,1.571) | 1.810(1.521,2.155) | 1.408(1.234,1.606) | 0.917(0.822,1.024) | 1.588(1.249,2.020) | 1.267(1.047,1.533) |
| Yan et al.2015 | 1.016(0.910,1.135) | 1.368(1.205,1.554) | 1.155(0.825,1.618) | 0.989(0.908,1.078) | 1.335(1.132,1.574) | 1.315(1.144,1.512) | 1.781(1.477,2.146) | 1.365(1.190,1.567) | 0.987(0.879,1.108) | 1.447(1.118,1.873) | 1.364(1.117,1.663) |
| Chatziste* et al.2014 | 0.973(0.857,1.106) | 1.377(1.213,1.563) | 0.966(0.634,1.471) | 0.964(0.885,1.050) | 1.253(1.072,1.465) | 1.337(1.158,1.544) | 1.827(1.536,2.174) | 1.381(1.202,1.586) | 0.946(0.831,1.076) | 1.600(1.260,2.032) | 1.297(1.073,1.568) |
| Atadzhanov et al.2013 | 0.982(0.865,1.116) | 1.386(1.223,1.570) | not included | 0.981(0.902,1.068) | 1.229(1.051,1.436) | 1.348(1.170,1.553) | 1.862(1.565,2.215) | 1.385(1.208,1.587) | 0.953(0.838,1.085) | 1.671(1.315,2.122) | 1.316(1.089,1.590) |
| Gelfand et al.2013 | 0.977(0.861,1.108) | 1.359(1.201,1.538) | not included | 0.979(0.900,1.065) | 1.223(1.047,1.429) | 1.336(1.160,1.539) | 1.818(1.528,2.162) | 1.365(1.193,1.563) | 0.951(0.837,1.081) | 1.626(1.281,2.063) | 1.290(1.067,1.559) |
| Balcerzyk et al.2010 | 0.979(0.862,1.112) | 1.379(1.217,1.563) | 0.971(0.640,1.471) | 0.979(0.899,1.065) | not included | 1.355(1.177,1.560) | 1.822(1.531,2.168) | 1.387(1.211,1.590) | 0.953(0.837,1.084) | not included | 1.283(1.062,1.551) |
| Tamam et al.2009 | 0.979(0.863,1.111) | 1.380(1.219,1.563) | not included | 0.977(0.898,1.063) | 1.234(1.056,1.442) | 1.341(1.164,1.544) | 1.843(1.550,2.191) | 1.382(1.206,1.583) | 0.951(0.837,1.081) | 1.633(1.287,2.072) | 1.306(1.081,1.578) |
| Tascilar et al.2009 | 0.987(0.868,1.122) | 1.379(1.216,1.563) | 0.988(0.647,1.510) | 0.979(0.900,1.066) | 1.254(1.072,1.466) | 1.347(1.169,1.552) | 1.820(1.529,2.166) | 1.386(1.209,1.589) | 0.960(0.843,1.094) | 1.586(1.248,2.015) | 1.285(1.063,1.554) |
| Wang et al.2009 | 0.984(0.863,1.123) | 1.356(1.198,1.536) | 1.009(0.649,1.569) | 0.972(0.891,1.060) | 1.161(0.984,1.371) | 1.309(1.141,1.502) | 1.792(1.504,2.135) | 1.353(1.184,1.546) | 0.960(0.841,1.097) | 1.598(1.255,2.035) | 1.294(1.069,1.566) |
| Lai et al.2007 | 0.990(0.871,1.125) | 1.376(1.213,1.561) | 1.036(0.687,1.562) | 0.975(0.897,1.062) | 1.239(1.060,1.450) | 1.333(1.155,1.537) | 1.852(1.557,2.202) | 1.375(1.198,1.578) | 0.958(0.841,1.091) | 1.649(1.299,2.093) | 1.316(1.089,1.590) |
| Parfenov et al.2007 | 0.997(0.879,1.130) | 1.375(1.213,1.560) | 0.986(0.649,1.499) | 0.988(0.908,1.075) | 1.243(1.064,1.452) | 1.339(1.161,1.545) | 1.833(1.541,2.181) | 1.375(1.199,1.577) | 0.971(0.856,1.101) | 1.608(1.267,2.042) | 1.301(1.076,1.573) |
| Kang and Lee.2006 | 0.983(0.864,1.118) | 1.366(1.205,1.548) | 1.008(0.666,1.527) | 0.974(0.895,1.061) | not included | 1.322(1.148,1.523) | 1.846(1.552,2.195) | 1.364(1.190,1.563) | 0.954(0.838,1.087) | not included | 1.314(1.087,1.587) |
| Gao et al.2006 | 0.985(0.866,1.119) | 1.368(1.207,1.549) | 0.983(0.647,1.495) | 0.980(0.901,1.067) | not included | 1.331(1.155,1.533) | not included | 1.369(1.195,1.568) | 0.958(0.842,1.091) | not included | not included |
| Baum et al.2006 | 0.980(0.861,1.115) | 1.377(1.213,1.562) | 0.933(0.618,1.410) | 0.985(0.904,1.073) | 1.232(1.053,1.441) | 1.348(1.168,1.556) | 1.817(1.528,2.162) | 1.382(1.204,1.586) | 0.956(0.838,1.089) | 1.609(1.267,2.042) | 1.285(1.063,1.553) |
| Pezzini et al.2005 | 0.982(0.864,1.117) | 1.365(1.205,1.548) | 0.961(0.635,1.454) | 0.982(0.902,1.069) | 1.234(1.056,1.441) | 1.328(1.152,1.530) | 1.830(1.539,2.175) | 1.366(1.192,1.567) | 0.958(0.842,1.091) | 1.621(1.278,2.056) | 1.300(1.076,1.570) |
| Cerrato et al.2005 | 0.980(0.861,1.115) | 1.401(1.239,1.584) | 0.955(0.626,1.457) | 0.983(0.902,1.070) | 1.239(1.061,1.448) | 1.372(1.195,1.575) | 1.863(1.565,2.218) | 1.407(1.232,1.608) | 0.954(0.837,1.087) | 1.620(1.277,2.055) | 1.301(1.075,1.573) |
| Jin et al.2004 | 0.986(0.867,1.121) | 1.362(1.202,1.544) | 0.984(0.645,1.500) | 0.982(0.902,1.069) | 1.231(1.053,1.439) | 1.320(1.147,1.520) | 1.836(1.544,2.185) | 1.361(1.188,1.559) | 0.960(0.843,1.093) | 1.632(1.286,2.071) | 1.311(1.084,1.584) |
| Duzenli et al.2004 | 0.992(0.874,1.127) | 1.391(1.230,1.572) | 1.010(0.661,1.520) | 0.986(0.905,1.073) | 1.236(1.058,1.444) | 1.355(1.180,1.555) | 1.842(1.549,2.190) | 1.396(1.221,1.595) | 0.963(0.846,1.095) | 1.632(1.287,2.071) | 1.298(1.074,1.570) |
| Slowik et al.2003 | 0.983(0.866,1.115) | 1.383(1.221,1.566) | not included | 0.979(0.900,1.066) | not included | 1.352(1.174,1.557) | 1.836(1.544,2.183) | 1.388(1.211,1.590) | 0.955(0.840,1.086) | not included | 1.300(1.076,1.570) |
| Souza et al.2003 | 0.991(0.873,1.125) | 1.394(1.232,1.577) | not included | 0.983(0.903,1.070) | 1.244(1.065,1.453) | 1.364(1.187,1.568) | 1.832(1.540,2.178) | 1.400(1.224,1.601) | 0.964(0.848,1.095) | 1.612(1.271,2.045) | 1.293(1.070,1.563) |
| Karttunen et al.2002 | 0.981(0.864,1.114) | 1.383(1.220,1.567) | 0.987(0.650,1.499) | 0.978(0.899,1.064) | 1.231(1.053,1.438) | 1.344(1.165,1.549) | 1.849(1.555,2.200) | 1.383(1.207,1.586) | 0.952(0.837,1.083) | 1.646(1.297,2.089) | 1.312(1.086,1.586) |
| Morrison et al.2002 | 0.982(0.862,1.120) | 1.379(1.212,1.569) | 0.997(0.655,1.518) | 0.977(0.897,1.064) | 1.218(1.036,1.430) | 1.345(1.161,1.557) | 1.851(1.547,2.215) | 1.381(1.120,1.590) | 0.956(0.837,1.091) | 1.682(1.312,2.155) | 1.301(1.068,1.583) |
| MacLeod et al.2001 | 0.979(0.861,1.113) | 1.395(1.232,1.580) | 0.973(0.642,1.477) | 0.977(0.897,1.064) | 1.241(1.061,1.451) | 1.366(1.187,1.572) | 1.853(1.557,2.205) | 1.401(1.224,1.604) | 0.950(0.834,1.082) | 1.641(1.292,2.084) | 1.304(1.078,1.576) |
| Chowdhury et al.2001 | 0.984(0.866,1.118) | 1.380(1.217,1.565) | 0.975(0.638,1.489) | 0.981(0.902,1.068) | 1.237(1.059,1.445) | 1.344(1.165,1.551) | 1.838(1.545,2.187) | 1.382(1.205,1.586) | 0.959(0.842,1.091) | 1.620(1.277,2.055) | 1.303(1.078,1.575) |
| Frikke-*t et al.2001 | 0.985(0.862,1.126) | 1.391(1.227,1.577) | 0.967(0.628,1.488) | 0.999(0.913,1.093) | 1.234(1.046,1.457) | 1.349(1.161,1.567) | 2.058(1.703,2.488) | 1.387(1.201,1.601) | 0.959(0.838,1.098) | 1.854(1.433,2.399) | 1.434(1.165,1.764) |
| Catto et al.2000 | 0.987(0.867,1.124) | 1.391(1.227,1.577) | not included | 0.984(0.903,1.072) | 1.254(1.072,1.466) | 1.358(1.177,1.567) | 1.875(1.574,2.233) | 1.395(1.216,1.600) | 0.959(0.841,1.094) | 1.630(1.281,2.073) | 1.313(1.084,1.590) |
| Kokubo et al.2000 | 0.958(0.850,1.079) | 1.388(1.224,1.574) | 0.856(0.604,1.211) | 0.975(0.896,1.062) | 1.231(1.053,1.439) | 1.353(1.172,1.562) | 1.862(1.564,2.216) | 1.393(1.215,1.598) | 0.936(0.826,1.061) | 1.652(1.301,2.098) | 1.317(1.089,1.592) |
| Peng et al.1999 | 0.987(0.869,1.122) | 1.364(1.204,1.545) | 0.998(0.657,1.514) | 0.980(0.900,1.067) | 1.233(1.056,1.441) | 1.326(1.151,1.526) | 1.831(1.540,2.178) | 1.364(1.191,1.562) | 0.960(0.844,1.093) | 1.623(1.280,2.059) | 1.304(1.079,1.576) |
| Ji et al.1998 | 0.977(0.860,1.110) | 1.362(1.202,1.543) | not included | 0.974(0.895,1.060) | 1.239(1.061,1.448) | 1.323(1.149,1.524) | 1.817(1.528,2.161) | 1.365(1.191,1.564) | 0.951(0.836,1.081) | 1.604(1.264,2.036) | 1.293(1.071,1.564) |
| Margagl* et al.1998 | 0.982(0.864,1.116) | 1.357(1.198,1.537) | 0.979(0.643,1.491) | 0.976(0.896,1.063) | 1.239(1.059,1.449) | 1.328(1.152,1.531) | 1.780(1.495,2.119) | 1.364(1.190,1.563) | 0.956(0.840,1.089) | 1.570(1.235,1.994) | 1.258(1.039,1.522) |
| Kessler et al.1997 | 0.976(0.859,1.109) | 1.375(1.212,1.560) | 0.968(0.637,1.470) | 0.971(0.892,1.058) | 1.233(1.056,1.441) | 1.341(1.161,1.548) | 1.812(1.522,2.157) | 1.380(1.202,1.584) | 0.949(0.833,1.080) | 1.600(1.260,2.033) | 1.283(1.064,1.552) |
| Hachinski et al.1996 | 0.981(0.863,1.114) | 1.371(1.209,1.554) | 0.995(0.654,1.513) | 0.976(0.896,1.062) | 1.236(1.058,1.443) | 1.335(1.158,1.540) | 1.825(1.534,2.170) | 1.374(1.198,1.575) | 0.953(0.837,1.084) | 1.621(1.277,2.056) | 1.296(1.072,1.566) |
| Couderc et al. 1993 | 0.985(0.867,1.120) | 1.390(1.227,1.574) | 0.986(0.647,1.502) | 0.981(0.901,1.068) | 1.236(1.058,1.443) | 1.357(1.179,1.564) | 1.840(1.547,2.189) | 1.394(1.212,1.597) | 0.957(0.841,1.090) | 1.622(1.279,2.058) | 1.300(1.075,1.570) |
| Qian et al.2012 | 0.981(0.864,1.114) | 1.368(1.207,1.550) | not included | 0.978(0.898,1.064) | not included | 1.338(1.161,1.542) | 1.821(1.531,2.166) | 1.373(1.98,1.574) | 0.954(0.839,1.086) | not included | 1.294(1.071,1.563) |
| Konialis et al.2016 | 0.991(0.873,1.126) | 1.359(1.200,1.539) | 1.001(0.660,1.519) | 0.979(0.900,1.065) | 1.224(1.048,1.430) | 1.326(1.151,1.528) | 1.821(1.531,2.167) | 1.360(1.187,1.557) | 0.965(0.848,1.097) | not included | 1.295(1.072,1.565) |
| Fayed et al.2009 | 0.976(0.860,1.108) | 1.355(1.200,1.531) | not included | 0.979(0.900,1.065) | 1.214(1.039,1.419) | 1.328(1.156,1.526) | 1.796(1.509,2.138) | 1.357(1.188,1.549) | 0.954(0.848,1.097) | 1.618(1.275,2.053) | 1.298(1.075,1.569) |
| Stankovic et al.2004 | 0.998(0.880,1.131) | 1.367(1.205,1.549) | 1.010(0.667,1.531) | 0.985(0.905,1.072) | 1.241(1.062,1.450) | 1.328(1.152,1.531) | 1.823(1.532,2.170) | 1.365(1.191,1.565) | 0.970(0.855,1.101) | 1.604(1.263,2.035) | 1.297(1.073,1.568) |
| Pedro-Botet et al.1992 | 0.980(0.862,1.114) | 1.364(1.204,1.546) | 0.959(0.634,1.450) | 0.980(0.898,1.064) | 1.241(1.062,1.449) | 1.324(1.150,1.524) | 1.823(1.532,2.170) | 1.366(1.192,1.565) | 0.957(0.840,1.089) | 1.606(1.265,2.038) | 1.306(1.080,1.580) |
| Fekih-M* et al.2014 | 0.984(0.867,1.117) | 1.362(1.203,1.541) | not included | not included | not included | 1.333(1.159,1.534) | 1.820(1.531,2.165) | 1.370(1.197,1.567) | 0.958(0.843,1.089) | not included | 1.297(1.073,1.567) |
| Brewin et al.2020 | 0.978(0.860,1.111) | 1.361(1.201,1.542) | 0.995(0.655,1.511) | 0.981(0.901,1.067) | 1.201(1.026,1.405) | 1.340(1.162,1.544) | 1.805(1.515,2.150) | 1.364(1.190,1.562) | 0.955(0.837,1.088) | 1.660(1.304,2.113) | 1.295(1.053,1.543) |
| Saidi et al.2009 | 0.975(0.858,1.108) | 1.347(1.193,1.520) | not included | 0.976(0.896,1.062) | 1.183(1.007,1.389) | 1.305(1.140,1.495) | 1.687(1.410,2.018) | 1.344(1.180,1.532) | 0.954(0.837,1.086) | 1.544(1.206,1.975) | 1.252(1.030,1.522) |
| Giassakis et al.2007 | 0.980(0.863,1.114) | 1.370(1.209,1.553) | not included | not included | not included | not included | not included | not included | not included | not included | not included |
| Nakata et al.1997 | 0.989(0.872,1.122) | 1.368(1.208,1.550) | not included | not included | not included | not included | not included | not included | not included | not included | not included |
| Szolnoki et al.2002 | 0.984(0.863,1.122) | 1.354(1.198,1.531) | not included | not included | not included | not included | not included | not included | not included | not included | not included |
| Aalto-Setala et al.1998 | 0.996(0.877,1.131) | 1.395(1.233,1.579) | not included | not included | not included | not included | not included | not included | not included | not included | not included |
| Artieda et al.2008 | not included | not included | not included | not included | 1.232(1.055,1.440) | not included | not included | 1.380(1.203,1.583) | 0.954(0.837,1.086) | not included | not included |
| Schneider et al.2005 | not included | not included | not included | not included | not included | not included | not included | 1.365(1.191,1.565) | not included | not included | not included |
| Li et al.2016 | not included | not included | not included | not included | not included | not included | not included | 1.371(1.195,1.572) | not included | not included | not included |

**Supplementary material Table 5. Publication bias and Heterogeneity of our meta-analysis.**

| Publication bias and Heterogeneity | Group | Value | Allele Comparisons | | Genotype Comparisons | | | | | ε4 carriers Comparisons | ε2 carriers Comparisons | ε4 homozygosis versus ε4 heterozygote | |
| --- | --- | --- | --- | --- | --- | --- | --- | --- | --- | --- | --- | --- | --- |
|  |  |  | ε2allele vs. ε3allele | ε4allele vs. ε3allele | ε2/ε2 vs. ε3/3 | ε2/ε3 vs. ε3/3 | ε2/ε4 vs. ε3/3 | ε3/ε4 vs. ε3/3 | ε4/ε4 vs. ε3/3 | ε4 vs. non-ε4 | ε2 vs. non-ε2 | ε4/ε4 vs. ε2/4 | ε4/ε4 vs. ε3/4 |
| Egger's test | All groups | P^Egger^ | 0.475 | 0.960 | 0.598 | 0.164 | 0.492 | 0.292 | 0.228 | 0.288 | 0.630 | 0.442 | 0.044 |
|  | Subgroups | P^Egger^ | 0.888 | 0.565 | 0.030 | 0.765 | 0.222 | 0.984 | 0.873 | 0.861 | 0.475 | 0.326 | 0.683 |
| Effect of heterogeneity | All groups | I^2^ | 65.40% | 77.80% | 50.20% | 45.10% | 12.00% | 68.90% | 38.90% | 74.90% | 58.20% | 23.80% | 0.00% |
|  |  | P^Q^ | ＜0.001 | ＜0.001 | ＜0.001 | 0.001 | 0.254 | ＜0.001 | 0.004 | ＜0.001 | ＜0.001 | 0.092 | 0.578 |
|  | Subgroups | I^2^ | 55.00% | 53.90% | 29.60% | 39.30% | 0.00% | 59.10% | 0.00% | 53.20% | 33.90% | 8.90% | 0.00% |
|  |  | P^Q^ | ＜0.001 | ＜0.001 | 0.073 | 0.014 | 0.891 | ＜0.001 | 0.802 | ＜0.001 | 0.031 | 0.338 | 0.882 |
